# Ensuring patient and public involvement in the transition to AI‐assisted mental health care: A systematic scoping review and agenda for design justice

**DOI:** 10.1111/hex.13299

**Published:** 2021-06-12

**Authors:** Teodor Zidaru, Elizabeth M. Morrow, Rich Stockley

**Affiliations:** ^1^ Department of Anthropology London School of Economics and Political Science (LSE) London UK; ^2^ Research Support NI Belfast UK; ^3^ Surrey Heartlands Health and Care Partnership Guildford and Waverley CCG Guildford UK; ^4^ Insight and Feedback Team Nursing Directorate NHS England and NHS Improvement London UK; ^5^ Surrey County Council Kingston upon Thames UK

**Keywords:** artificial intelligence, big data, design justice, digital health technology, machine learning, mental health, patient and public involvement, public engagement, scoping review

## Abstract

**Background:**

Machine‐learning algorithms and big data analytics, popularly known as ‘artificial intelligence’ (AI), are being developed and taken up globally. Patient and public involvement (PPI) in the transition to AI‐assisted health care is essential for design justice based on diverse patient needs.

**Objective:**

To inform the future development of PPI in AI‐assisted health care by exploring public engagement in the conceptualization, design, development, testing, implementation, use and evaluation of AI technologies for mental health.

**Methods:**

Systematic scoping review drawing on design justice principles, and (i) structured searches of Web of Science (all databases) and Ovid (MEDLINE, PsycINFO, Global Health and Embase); (ii) handsearching (reference and citation tracking); (iii) grey literature; and (iv) inductive thematic analysis, tested at a workshop with health researchers.

**Results:**

The review identified 144 articles that met inclusion criteria. Three main themes reflect the challenges and opportunities associated with PPI in AI‐assisted mental health care: (a) applications of AI technologies in mental health care; (b) ethics of public engagement in AI‐assisted care; and (c) public engagement in the planning, development, implementation, evaluation and diffusion of AI technologies.

**Conclusion:**

The new data‐rich health landscape creates multiple ethical issues and opportunities for the development of PPI in relation to AI technologies. Further research is needed to understand effective modes of public engagement in the context of AI technologies, to examine pressing ethical and safety issues and to develop new methods of PPI at every stage, from concept design to the final review of technology in practice. Principles of design justice can guide this agenda.

## 1 INTRODUCTION

Machine‐learning algorithms and big data analytics will revolutionize contemporary health care. Popularly described as ‘artificial intelligence’ (AI), these technologies are being used in health‐care systems across the globe, for example to process population data and identify at‐risk groups, or to determine the best treatment options for individual patients, and to develop precision medicine.^1^, ^2^

Despite the advantages of efficiency of scale and depth of computational power,^3^, ^4^, ^5^ concerns have been expressed by scientists, practitioners and broader publics about the systematic datafication of people's lives and their lived experiences of health and illness.^6^, ^7^, ^8^, ^9^, ^10^ It is unclear whether AI‐assisted health care always leads to better patient outcomes, whether it empowers and enables patients/service users, carers and their families, and whether patients or the public have a meaningful say over AI‐assisted processes of care or design of such systems.^11^, ^12^, ^13^

This paper explores the issues from the perspective of ensuring that patient and public involvement is not overlooked in imaging and transitioning to AI‐assisted health care. There are implications here for the values of equality, diversity and inclusion in a human/digital intelligent world, which there is only limited space to touch upon.

In medicine and health, patient and public involvement (PPI) has become a principle for health‐care providers and a field of practice and research. In different countries, alternative terms include personal and public involvement (P&PI) or patient and public engagement (PPE). Across the globe, there are major institutions that support PPI, including National Health Service (NHS England) patient and public participation frameworks, the UK National Institute for Health Research Centre for Dissemination and Engagement, the US Patient‐Centered Outcomes Research Institute (PCORI)^14^ and the National Health and Medical Research Council in Australia. Previous research on PPI in health has drawn attention to issues of equality, diversity and inclusion (EDI) and the professional dominance of the PPI agenda.^15^, ^16^, ^17^, ^18^, ^19^ However, this body of work has yet to contend with the urgent issues^20^, ^21^, ^22^ of how PPI might be developed in an AI‐assisted health and research system where ‘unexplainable’ decisions are being made by computers and technology designers.^22^, ^23^, ^24^, ^25^

New forms of interdisciplinary collaboration^26^ between patients, designers, data scientists, clinicians, researchers, computer scientists, developers and entrepreneurs are emerging, but very slowly and disproportionately to the scale and speed of technological change. They aim to create innovative, user‐validated and socially responsible products and e‐services with the people who stand to benefit from them, not only with specialists, or health professionals whose views are known to differ from patient perspectives.^27^, ^28^ Advances have been made in participant co‐design theory and methods,^29^, ^30^, ^31^ such as the Design Council's (UK) Double Diamond methodology, and in participative medical device design.^32^, ^33^ New ‘social licences’ for digital technologies,^34^ new guidance such as the World Health Organization's mHealth Evidence and Assessment (mERA) checklist and a push for ‘explainable AI’ (XAI) highlight the need to improve the quality and consistency of user‐centred and more inclusive technology conception and design processes.^35^

This paper draws together evidence about public engagement in the context of newly emerging AI technologies for health to inform new strategies for PPI in health care. The concept of design justice provides a useful perspective that promotes engagement^16^, ^36^ and aims to explicitly challenge exclusion and inequalities by valuing inclusion and diversity in design.^17^, ^19^, ^37^, ^38^, ^39^

## 2 AIMS AND APPROACH

We chose to focus our exploration on mental health as this is an area of care where the take‐up of big data and machine‐learning software has already gathered significant pace, and often without public debate on what desirable safeguards should be put in place.^1^ Machine‐learning applications in clinical psychology and psychiatry are appealing as cost‐cutting sources of scientific knowledge and evidence‐based policy in public mental health care.^40^, ^41^ This trend is very likely to accelerate as the COVID‐19 pandemic worsens the mental health crisis and digital solutions become available to triage patients, address the ‘backlog’ to care and to expediate treatment or interventions.^42^, ^43^ Depending on the direction of development, AI could also have major benefits for smoothing patient pathways, enhancing workflow in health systems, regulating quality and enabling quality improvement of care.

As this is a new and rapidly evolving context for PPI, we did not want to be too narrow in our view of what PPI is or could become. In the UK context, PPI is at present defined in government policy as a requirement for all publicly funded research and health care. We drew upon existing definitions (to develop our search terms) while also exploring a wider notion and the broader landscape of ‘public engagement’ to allow possibilities for new modes and opportunities for PPI to be identified in the literature.

The approach was therefore to look broadly at evidence on patient participation, patient perspectives, approaches to co‐production and user‐led projects, as well as patient engagement in clinical care, care delivery and service design. We included patient engagement with AI technologies in health‐care contexts and in self‐management of mental health conditions and personal well‐being as these are important ways that patients are engaging with AI technologies and mental health care.

The review explored the following questions:

- What are the main issues and challenges associated with data‐driven AI‐assisted care that public engagement might help to address?
- How and in what contexts have patients and the public been involved in the design of AI technologies in mental health?

**FIGURE 1:**
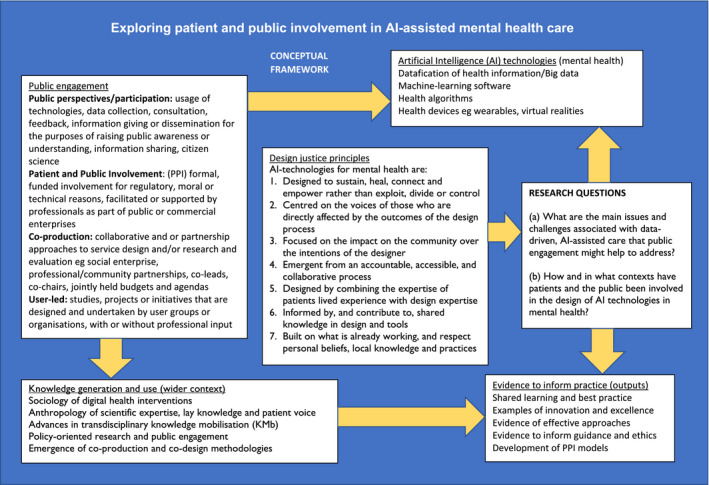
Exploring patient and public involvement in AI‐assisted mental health care

Our approach was informed by a conceptual framework, illustrated by Figure 1, which draws on design justice perspectives (described below), the sociology of digital health interventions,^44^, ^45^ the anthropology of scientific expertise^46^ and advances in transdisciplinary knowledge mobilization (KM), all of which can inform policy‐oriented research and public engagement through co‐production or co‐design methodologies.^47^ These areas of practice and expertise share a concern with attending to the uses of knowledge^48^ and its conditions of possibility: how it is produced, for what purpose, the type of knowledge produced, about what or whom and on whose terms.^49^ The framework enabled us to draw on these perspectives to inform the methods described below.

## 3 METHODS

We used a systematic scoping review as this would allow us to focus on identifying issues and themes across an emerging topic field and draw together evidence from relevant published literature, including, but not limited to, evidence about potential avenues for PPI.^50^, ^51^

We sought information about how machine‐learning and data‐intensive technologies might enable meaningful and effective public engagement, as well as clarification on how patients and broader publics can contribute to the design of such technologies through a formal PPI process.

### 3.1 Inclusion/Exclusion

We sought evidence from research studies and insights from professional or patient expertise about public engagement in AI‐assisted mental health. Included articles were those that mentioned or reflected on: (a) any type of patient and public groups involved in one capacity or another, for example as users of technologies, research subjects, public reviewers, patient representatives or co‐researchers; (b) contexts of involvement in the design process; (c) approaches to involvement, ranging from reports on user engagement, to user testing, interviews, consultations, participatory design concepts and shared decision‐making practices; (d) evidence of influence or impact of involvement on design decisions, practices or outcomes, such as published case studies of impact or evaluation reports that explain how a PPI element influenced a research study or the design of a health intervention; and (e) patient and public views on data‐driven approaches to mental health care and research. We sought journal articles, conference proceedings and grey literature. We excluded articles published before 2010, articles published in other languages and articles that did not relate to mental health.

### 3.2 Search strategy

We used Web‐based searches (carried out in December 2020) through Web of Science (all databases) and Ovid (MEDLINE, PsycINFO, Global Health and Embase). The electronic searches were supported and enhanced with handsearching to identify relevant references outside of the research literature. We hand‐searched the journal Health Expectations, the INVOLVE database and materials published by mental health charities. Forward searches were used to track citations of key articles as they are referenced in new articles. Backward search involved looking through the reference lists of returned articles to identify highly relevant source references. We retrieved documents through library search engines, which index published materials and conference proceedings. We also made use of the PCORI Engagement in Health Research Literature Explorer.

### 3.3 Key search terms

Searches used terms associated with ‘public engagement’ used by Brett and colleagues^18^ in their systematic review of the impact of PPI and terms used by PCORI. We developed composite terms for AI technologies derived from the literature (see Table 1). Database‐specific MeSH terms for mental health (and other terms where available) were used to expand and consolidate the searches. The full searches can be made available upon request.

**TABLE 1:** Key search terms and medical subject headings (MeSH)

Concept	Patient/public concept	Engagement concept	AI technologies concept	Mental health concept
MeSH terms	Community participation Patient participation Stakeholder participation	Patient engagment Patient experience Patient and public involvement Patient consultation Citizen science Community involvement Client participation	Affective computing Algorithms Artificial intelligence Artificial neural networks Automation Bioinformatics Computer‐Assisted Data Mining Decision support systems Deep Learning Digital health Diagnosis Electronic health record Human machine systems Information systems Machine learning Medical Informatics Pattern recognition	Mental disorders Mental health (mental health research, mental health practice, mental health care, mental health illness assessment) Mental stress Psychiatric (psychiatric patients, psychiatric care, psychiatric symptoms) Psychological (psychological diagnosis, psychological assessment, psychopathology)
Key terms	Caregiver(s) Carer Client Clinician(s) Citizen Community(ies) Consumer(s) Employer(s) Family Hard‐to‐reach/Hard to research Insurance Lay Partner(s) Patient(s) Payer People (older people, younger people) Pharmaceutical Policy makers Provider(s) Public Relative Stakeholder(s) Seldom‐heard Survivor User (service user, service users, user‐led) Vulnerable	Advisory board Centre/center* (centred, centered, center, centre) Collaborat* (collaborator, collaboration, collaborate, collaborative) Consult* (consultation, consulted, consultation) Consumer panel Co‐produc* (co‐produced, co‐ Co‐design production) Engage* (engagment, engaged, engaging) Evaluat* (evaluation, evaluate, evaluator) Focus group Input Interview Involvement (patient and public involvement, public involvement) Particip* (participate, participant, participating) Partner* (partner, partnership) Perspective Service transformation Service design Voice	Algorithm (health algorithm, algorithm software) Big data Chatbot Data mining Deep learning Digital technology Digital eHealth/e‐health electronic health Health technolog* (health technologies, health technology) Medical device Neural network Natural language processing mHealth Wearable Smartphone Virtual (virtual reality)	

### 3.4 Data extraction

**FIGURE 2:**
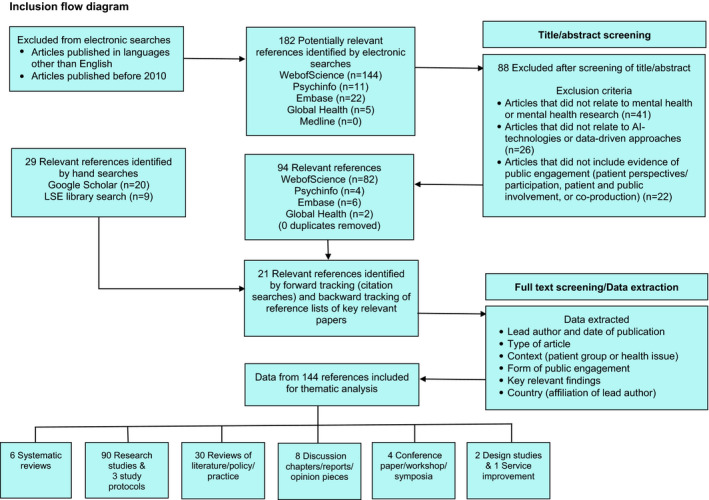
Flow diagram of inclusion criteria

The titles/abstracts of 182 identified articles were read, and if deemed to be relevant to the aim of the review, they were retrieved in full for analysis. The screening process is illustrated by an inclusion flow diagram (Figure 2).

### 3.5 Analysis

We explored and identified themes in the data using a series of thematic analysis techniques^52^ supported by the use of summaries of articles (literature tables) and analytic tools within NVivo. First, we iteratively developed a thematic code framework to draw out^53^ emerging themes and subthemes, while retaining links to the original sources to aid retrieval.^52^ Next, seven principles of design justice (drawing on the international Design Justice Network's ten principles) (see Figure 1) for AI technologies for mental health were applied as a lens through which to consider how AI technologies are currently being used, and as a perspective to identify issues for future research to address. We considered these principles against the themes in the literature, particularly in relation to PPI in the development and use of AI technologies.

### 3.6 Rigour

The review method was developed and revised by all team members, including identification of databases to be searched and key search terms. Strategies for minimizing biases in the search strategy were as follows: (a) two team members independently cross‐checked more than half of the returned papers against included/exclusion criteria; (b) members of the team discussed and reached agreement on the thematic code framework; and (c) inclusion and use of grey literature extended the searches beyond peer‐reviewed articles.

As part of the approach, we reflected on the strengths and limitations of our perspectives as an anthropologist (INITIALS1), research analyst/inclusion advocate (INITIALS2) and social researcher working in an integrated care system (INITIALS3). These techniques included critical reflective conversations about the meaning of PPI, knowledge mobilization and AI technologies in our respective areas of interest and expertise. These conversations allowed us to challenge each other by identifying assumptions in our ways of conceptualizing public engagement, and to use co‐authorship to cross‐check the resonance of our arguments with the evidence.

A preliminary version of this review was presented for critical appraisal at UCL's QHRN (Qualitative Health Research Network) workshop on 11 September 2020. Resulting conversations shaped our understanding about the complexity of data sharing/ownership within health‐care systems and the importance of transparency to service users.

## 4 RESULTS

All 144 articles included in the scoping review are summarized in literature tables in Appendix 1. Studies mostly originated from the USA, UK, Australia, New Zealand and the Netherlands. It is likely that our searches did not pick up publications relating to forms of ‘public engagement’ in China, and our search terms should be considered in the light of their Western origins. Overall, there was little evidence of work that pursued or engaged in PPI, or patient involvement (5 articles). Nevertheless, the literature does reveal multiple modes for engagement of users, including the following:

Design as inclusive practice, for example experts by experience and user representatives within design teams as communities of practice^54^
Patient‐focused research networks, research collaborations and projects, for example patient‐practitioner‐researcher collaboration^55^
User‐focused design practices, for example workflow analysis, user feedback,^56^ user experience design (UX design) or testing, and ‘engagement checks’ within a user‐centred approach to engage specific user groups in co‐design^57^
Experience‐led design or research methods, for example experience‐based co‐design (EBCD)^58^; and theory‐driven approaches, for example user‐centric explainable AI (XAI)^35^
Qualitative research, for example methods to capture patient or user perspectives, including focus groups, patient interviews, ethnography and observation, and semi‐structured interviews^59^
Quantitative methods, for example patient surveys, rating scales and PROs
Consensus building, for example Delphi methods^60^
Involvement of communities or social networks in decision making or the provision of care.^61^

These approaches have mainly been used to access patient and public perspectives and use them in a manageable way to inform design practices, for example statistics, stories, vignettes and case studies. Many studies indicated that direct public involvement in decisions about technological design can drive innovation while offering a moral means of patient empowerment.^54^, ^62^, ^63^, ^64^, ^65^, ^66^, ^67^, ^68^, ^69^, ^70^, ^71^, ^72^, ^73^, ^74^, ^75^, ^76^

Some studies used data‐intensive technologies as a means for engaging marginalized service users and mobilizing their experiences to develop new structures of public accountability and ways of rebuilding trust.^77^, ^78^ Yet, other articles reported on user‐led initiatives or responses to data‐driven applications in contemporary mental health services.^79^, ^80^ Other articles described the views of clinicians, patients and wider communities on data sharing, data protection or the use of smartphones,^72^ smart fabrics and wearables,^81^, ^82^ social media and machine‐learning algorithms for managing and monitoring mental distress.^83^, ^84^, ^85^, ^86^, ^87^

A few articles described involving patients and other stakeholders as experts by experience, expert consultants and respondents in an effort to evaluate the acceptability and feasibility of research^14^ or data analytics in mental health care.^59^, ^88^, ^89^, ^90^, ^91^, ^92^, ^93^, ^94^, ^95^, ^96^, ^97^, ^98^, ^99^ Others showed commitment to user engagement or user experience (XE) to enhance randomized controlled trials,^100^ implementation reviews, guidelines for design or reviews of commercially available mental health apps.^101^, ^102^, ^103^, ^104^, ^105^, ^106^, ^107^, ^108^, ^109^, ^110^, ^111^, ^112^

Thematic analysis identified three major themes in the literature (summarized by Table 2) that reflect the challenges and opportunities associated with AI‐assisted mental health care:

1. Applications of AI technologies in mental health care
2. Ethics of public engagement in AI‐assisted mental health care
3. Public engagement in the planning, development, implementation, evaluation and diffusion of AI technologies

**TABLE 2:** Thematic analysis

Theme 1: Applications of AI technologies in mental health care	
Subthemes	Issues
Assessment and observation (25 articles)	Using data‐driven applications for clinical assessments by monitoring or observing mental distress^26^ Complex ecologies of intersecting data streams and multiple collection points, for example smartphones, wearable biosensors and social media,^65^, ^93^, ^113^, ^116^, ^117^ through electronic health records,^114^ patients using ‘mHealth’ devices ^115^ Real‐time/momentary assessment of mental distress^118^, ^119^ and iHealth^118^ Limitations of eHealth^120^ Tracking and predicting relapses in severe mental disorders^65^, ^69^, ^94^, ^97^, ^98^ Observing and measuring mental health distress and identifying at‐risk individuals by processing ‘naturally’ occurring linguistic data (natural language processing and sentiment analysis)^121^, ^122^, ^123^, ^124^, ^125^ Understanding types of big data, for example syntax, semantics and speech acoustics, location, biomarkers and technology usage^126^, ^127^, ^128^, ^129^
Diagnosis (6 articles)	Decision support tools^130^ Detecting error and understanding diagnostic accuracy^131^, ^132^ Complexity and accuracy of ‘ground truths’ on which algorithms are based^1^ Choosing the right type of machine‐learning algorithm^87^ Recognizing the limitations of the source data for accurate algorithms/machine learning and selecting the best indicators and combinations of biomarkers for complex mental health disorders^204^
Treatment/therapy (40 articles)	Therapeutic potential of data‐rich environment, and increased self‐awareness and self‐management of mental health^62^, ^86^, ^117^, ^133^, ^134^ plan ahead to avoid crisis^135^ or prevent suicide^136^ Access to mental health data whenever and wherever professionals/patients choose^62^, ^86^, ^117^ Access and communication in immersive/virtual reality clinical environments^64^, ^93^, ^95^, ^137^, ^138^ Potential adverse effects or consequences of virtual clinical therapies on patients^91^, ^139^ Failure to recognize the need for stakeholder engagement in design processes^92^, ^104^, ^108^, ^109^ Disconnect between the interests of private developers, with research evidence, professional expertise and patient experience^92^, ^104^, ^108^, ^109^ Effectiveness of digital therapies for long‐term resolve of mental health conditions^102^ and carer support^140^, ^141^, ^205^ Design of therapies based on digital devices rather than patient need^102^ Uncertainties about which digital therapies work best for whom, when and why^54^, ^103^, ^104^, ^106^ Creating AI technologies for culturally diverse communities, for example overcoming language barriers, disability or communication challenges^54^, ^103^, ^104^, ^106^ Differences in the quality of engagement with patient support networks, formal carers and family carers in AI‐assisted therapy^78^, ^80^
Integration/ personalization of care (19 articles)	The role of AI technologies in integration of health and social care services^71^ Recording and combining multiple types and sources of patient data^110^ Treating mental and physical illnesses, situating patient experience across different care services or understanding the connections between health and everyday life more broadly^59^, ^64^, ^77^, ^117^, ^206^ The benefits of understanding patient care across multiple services and service settings^68^, ^73^, ^90^, ^142^, ^143^ Different patient preferences for gathering and visualizing care received and care plans^66^, ^70^, ^72^ Different patient preferences for data linkages and transfer, for example social media accounts and electronic medical records^99^, ^144^ Challenges of collecting, collating and interpreting data from multiple sources^59^ Engaging patients in work to interpret and contextualize data^21^ Uncertainties about data ownership and responsibilities for personal data protection between individuals, organizations and institutions^56^

Theme 2: Ethics of public engagement in AI‐assisted mental health care	
Subthemes	Issues
Inequalities and population biases (14 articles)	Risk of AI technologies exacerbating social inequalities, for example disadvantaging people because of their gender, ethnicity, age, socio‐economic background, reproducing population biases or geopolitical inequalities^97^, ^111^, ^116^, ^146^, ^207^ Underserved and indigenous populations^146^, ^147^ The reliability of ground truth data and exclusion of seldom heard groups/people who do not access services^116^, ^148^ User‐centred methods to help to engage diverse and representative stakeholder groups in design of technologies or their use^56^, ^77^, ^111^ Lack of scrutiny about included/excluded groups in user experience design (UX design)^105^, ^149^, ^150^
Socio‐political context (11 articles)	Lack of critical and reflective public debate on the broader socio‐political context and digital technologies^40^, ^41^ New awareness of legal regulations, data risk assessment and PPI^158^ Tools that are culturally relevant^153^, ^154^ and culturally safe^155^ Maintaining public services in the context of growth of commercial/private providers of digital technologies^26^, ^151^ Misperceptions that digital technologies replace professional/medical advice or make it unnecessary^152^ Looking at the broader contexts/environments of people's lives, for example urban design^156^ and access to open spaces^157^
Safety and acceptability (21 articles)	Lack of safety measures, oversight and scrutiny^56^, ^159^ Uncertainty about public acceptability of basing mental health‐care interventions upon predictive analytics^160^ Uncertainty about how digitally mediated communication might affect trust between patients and mental health professionals^26^ Uncertainty about how best to discuss the complexities of data quality, privacy and data protection issues with patients^85^, ^116^, ^161^ How to approach informed consent as a dynamic, on‐going and relational process, instead of a one‐off event^114^, ^161^, ^162^, ^163^ Professional roles and responsibilities for ensuring and communicating data protection safeguards^105^, ^106^, ^111^ Variations in public understanding of cybersecurity in relation to mental health data^84^ Diversity of patient's attitudes towards sharing mental health data^838560^ Digital teaching resources or experiences that hold authenticity and real‐world relevance^63^, ^164^, ^165^ Strength of public opinion that mental health apps should be developed collaboratively with patients^79^

Theme 3: Public engagement in the planning, development, implementation, evaluation and dissemination of AI technologies	
Subthemes	Issues
New contexts and opportunities for PPI (34 articles)	Rise in internet‐based trials^166^ Patients taking a more active role in their care through self‐monitoring are more aware and informed about the potential for health technologies^62^, ^78^, ^84^, ^152^, ^167^ Digital technologies are an avenue for including patient's experiential insights in research^63^ Patients using digital technologies are more motivated to maintain adherence to treatment^97^, ^168^ Digital approaches to support outside of therapy^169^ Digital technologies may facilitate engagement with clinicians, other patients, or friends and family^59^, ^72^, ^106^ Digital technologies may facilitate the involvement of informal caregivers in severe mental health disorders^118^ Patients using digital technologies to give feedback about service quality^77^ and patient‐reported outcomes^170^ Engaging underserved populations who are challenging to reach or engage for sustained periods of time^73^, ^93^, ^146^ Virtual reality scenarios in therapeutic contexts^171^, ^172^ Factors negatively affecting engagement^101^, ^174^ Challenges of quality assurance,^104^ implementation,^173^ usability^175^ and silos of innovation^176^ Ways of engaging with children and young people^67^, ^177^, ^178^, ^179^, ^180^, ^181^
Public awareness, preference and choice (14 articles)	Helping patients access high‐quality health information^182^ Diversity of patient attitudes, expectations and preferences towards active engagement^183^ and passive data collection in monitoring health and self‐management^62^ Digital technologies may offer greater choices about care, for example remote access to services and at flexible times^62^ Service users and clinicians prefer some technologies over others depending on features, functionalities or approaches (eg game‐based approaches^59^, ^72^, ^78^, ^103^, ^184^ real‐world stories or problem‐solving tasks^73^ Adaptability of systems to variability in individual preferences^65^ and non‐compatible preferences^103^ Availability of a wide variety of design choices makes it possible to tailor digital mental health technologies to different socio‐cultural contexts of use^66^, ^73^, ^104^, ^186^
Patient and public trust (21 articles)	Designing new technologies in a context of patient/professional trust,^187^ mental health stigma and fears of self‐disclosure^59^, ^95^ Data‐intensive technologies as a way to support disclosure of sensitive information, for example chatbots^95^ Anonymous feedback tools, for example on quality of care provided or across multiple providers^77^ Patients show willingness to trust and use technologies^188^ Complicating or compromising patient/carer/professional trust^26^, ^152^ The impact of initial beliefs about digital health technologies on engagement with interventions^189^ Interpersonal competencies, ‘bond’^190^ and inspiring feelings of fondness^191^ Barriers to data‐intensive technologies in mental health, including the impact of inequalities^97^, ^111^, ^116^, ^146^, ^193^ Building public trust through transparency about data ownership, privacy and data securityy^83^, ^84^, ^85^, ^160^, ^192^

### 4.1 Applications of AI technologies in mental health care

#### 4.1.1 Assessment and observation

Our findings show widespread interest in developing data‐driven applications in mental health care as tools for ‘eHealth’ (or e‐health, electronic health) including conducting clinical assessments and monitoring and observing mental distress more widely.^26^ Online clinical assessment and continuous observation is increasingly possible at a distance from patients,^113^ using electronic health records^114^ and an ecology of intersecting data streams collected through ‘mHealth’ (mobile health) devices.^115^ Such devices include smartphones and wearable/handheld biosensors, social media and other Web‐based activities.^65^, ^93^, ^116^, ^117^

The notion of ‘iHealth’ (intelligent health) further builds on and expands eHealth by using real‐time self‐monitoring within the patient's environment together with data processing and data mining to support personalized decision making.^118^ Though the evidence base is still in its infancy, forms of iHealth have been shown to be effective for identifying, monitoring and anticipating mental distress.^118^, ^119^ Current limitations to iHealth and eHealth more generally include financial costs, cultural, language and literacy barriers, power supply issues (eg in remote communities), data security and privacy issues.^120^

Feasibility studies in clinical psychiatry and biomedical engineering have shown that measuring markers of clinical status through data analytics can enable patients and clinicians to pick up early warning signs and accurately track and predict relapses in persons living with severe mental disorders.^65^, ^69^, ^94^, ^97^, ^98^ Similarly, recent work in affective computing and computational linguistics suggests that anxiety, depression, bipolar disorder and suicidal intent can all be observed and measured through data on ordinary ‘naturally’ occurring linguistic forms, such as posts and comments on social media platforms.^121^, ^122^, ^123^, ^124^, ^125^ Regardless of the source and type of data, it appears that everything from syntax (eg hashtag analytics of #depression on Instagram),^126^ semantics and speech acoustics to smartphone typing dynamics, step counts or UV light exposure might, eventually, be used to accurately detect and monitor mental distress.^127^, ^128^, ^129^

#### 4.1.2 Diagnosis

AI technologies have the potential to be effective clinical decision support tools (DST).^130^ Although augmenting, and in some cases exceeding, clinicians' abilities, the diagnostic accuracy of data analysis techniques such as machine‐learning algorithms is never a given and has been known to produce clinically unacceptable rates of error.^131^, ^132^ In mental health, reliability is a particularly challenging issue. ‘Ground truths’ (the sample data set machine‐learning systems are trained on) are difficult to establish because historically, mental health disorders have largely been defined by subjective and clinical features.^1^ The literature describes multiple classes of machine‐learning algorithms, each with their specific properties and varying advantages or limitations depending on the task they are used for and the data they are trained on,^87^ but public engagement is rare.

#### 4.1.3 Treatment/therapy

Data‐intensive technologies hold clear therapeutic potential. The abundance of digital data has facilitated pharmacoepidemiology and, in particular, observational research on the effectiveness of real‐world medication.^133^ Websites, social networking and smartphone apps^134^ are allowing persons experiencing mental distress to self‐manage, access information about their conditions whenever and wherever they choose^62^, ^86^, ^117^ and plan ahead to avoid crisis^135^ or prevent suicide.^136^

There are indications that therapies based on co‐designed immersive virtual reality environments^137^, ^138^ and virtual humans (eg chatbots) can overcome communication barriers and widen access to better quality care.^64^, ^93^, ^95^ However, it is difficult to distinguish between overzealous claims and actual evidence of therapeutic efficacy. Adverse effects and unintended consequences have been reported.^91^, ^139^ Caution has been widely expressed to the pressing need for therapists, clinicians, patients and the public to be systematically involved in design of therapeutic interventions (eg an abundance of mental health apps), which tends to be controlled by private software developers and commercial providers whose products are often disconnected from empirical evidence, professional expertise in mental health care, or the lived experience of patients.^92^, ^104^, ^108^, ^109^ There is a recognized need to involve culturally diverse communities who speak multiple languages, people with disabilities and communication challenges, support networks of formal carers and family carers,^140^, ^141^ all of whom can play an active role in formulating guidelines and standards for evaluating safety and patient outcomes.^54^, ^103^, ^104^, ^106^

#### 4.1.4 Integration/personalization of care

The use of patient‐generated data in mental health care is limited, but not for much longer. By 2040, AI technologies are expected to play a major role not just in the provision of care but also in integrating health and social care systems, certainly in the UK.^71^ This makes sense given that recording and combining multiple types of data tends to increase diagnostic accuracy,^110^ improve personalization of care, treat comorbid mental and physical illnesses, track patient experience across different care services and understand the connections between health and everyday life more broadly.^59^, ^64^, ^77^, ^117^ Gathering and visualizing data through digital technologies for integration in care services and individual care plans consistently comes up as desirable in co‐design activities.^66^, ^70^, ^72^

Systems of data integration may seem intuitive to patients with comorbidities, as they do not tend to explicitly separate mental and physical health needs,^64^ or see their experiences of health and illness as a separate part of their lives. A large proportion of research participants recruited in an emergency care setting (over 70 per cent) have agreed to have their social media activity linked with their electronic medical records, though agreement was higher in younger social media users.^99^ Kumar outlines a framework involving mental health practitioners and various stakeholders at different levels and the channels in which technology can be leveraged while keeping the patients' rights front and centre.^142^ A perspective that has not yet been given due attention is the integration of AI technologies outside of clinical settings^67^, ^143^ or in the everyday spaces where comorbid conditions and habits inter‐relate.^68^, ^144^

How data are or could be collected, collated and interpreted marks a common challenge for integrating digital technologies in care services^59^ and interpreting data.^21^ However, the toughest challenge for digitally enabled integrated care is in the complexities associated with data ownership, the economic value of personal data, and the monetization of data or ‘knowledge exchange’ practices between various companies and institutions. Public engagement in such decisions could play a critical role in earning public trust at the intersection of competing private and public interests. It could also inform strategies to ensure the interoperability of digital mental health technologies with electronic health records^56^ and other forms of personal digital data.

### 4.2 Ethics of public engagement in AI‐assisted mental health care

#### 4.2.1 Inequalities and population biases

Using AI technologies for the early detection of mental health concerns and improving access to evidence‐based interventions have great potential to lead to improved health outcomes,^145^ particularly for underserved, minority or indigenous populations.^146^, ^147^ However, the literature is full of evidence that data‐driven approaches to mental health care can further entrench gendered, ethnic, racial, age‐based, class‐based and geopolitical inequalities.^97^, ^111^, ^116^, ^146^ The primary concern is that if the ground truth data are limited to social media data or self‐reported medical diagnoses of mental health status, the data will produce biased results: for example, insights will only be generalizable to digitally literate groups or individuals who have sought professional care and felt secure enough to disclose their mental health status.^116^ Most algorithms reproduce and overlook data biases associated with the sex and gender dimension and its contribution to health and disease differences among individuals.^148^ Involvement of people with learning disabilities is consistently overlooked as being too complex, meaning there are very few digital interventions developed with these groups.^149^ In effect, those on the losing side of the digital and socio‐economic divides are further alienated, excluded or disengaged from receiving the care they need.^56^, ^77^, ^111^, ^150^

#### 4.2.2 Socio‐political context

Public engagement on AI‐assisted mental health care will need to include critical and reflective debates on the broader socio‐political context and its influence in shaping professional practice and treatment of mental disorders.^40^, ^41^ There are concerns that maintaining public service provision at the forefront of policymaking and technological development will be an uphill struggle, but this remains an open question.^26^, ^151^ Furthermore, there is evidence that ideals of individual responsibility and self‐help can mislead service users into believing that digital technologies yield infallible and objective knowledge of mental distress.^152^ Misplaced trust in technology could lead to people not seeking professional help when they need it. Valuing principles of diversity and inclusion in technology design is important here because the design of digital tools and interventions must be based on a deep knowledge of the subtleties that distinguish between the social and cultural contexts of use,^153^, ^154^, ^155^ which include the environments and infrastructures where people live.^156^, ^157^

Imagining new digitally enabled contexts for care requires practitioners to have awareness of emerging data/legal regulations, data risk assessment and effective strategies for patient engagement, within a necessary medical‐ethical framework for innovation in health‐care technology.^158^

#### 4.2.3 Safety and acceptability of new practices

Numerous articles point to uncertainty and signal that machine‐learning algorithms, big data and associated digital technologies are placing mental health researchers and care providers in ethically uncharted territory where little is known about safety and acceptability,^159^ for example issues of how and whether to base care interventions upon predictive analytics,^160^ what digitally mediated communication might hold for therapeutic relationships between patients and mental health professionals,^26^ and how to discuss privacy and data protection with patients when the clinically ideal data set is also the most intrusive, and contend with the issue of why the most insightful algorithms are often the ones whose reasoning cannot be accounted for.^85^, ^116^, ^161^

Given the importance of the ethical and moral alignment issues at stake, raising public awareness and understanding of the pros and cons of AI ‐technologies is essential. Failing to involve patients and the public could lead to innovations or applications that are considered unacceptable, are publicly criticized and finally withdrawn. This rejection of AI is exemplified in the public response to the Samaritans Radar, a Twitter app for suicide prevention that failed to engage with the community that it was designed for.^79^

In the case of mental health apps, leaders in mHealth research, industry and health‐care systems from around the globe are seeking to promote consensus on implementing standards and principles for their evaluation.^56^ Emerging guidelines indicate two promising strategies for safety and acceptability. One is the rethinking of informed consent in the context of AI technologies as a dynamic, on‐going and relational process, instead of a one‐off event^114^, ^161^, ^162^, ^163^ The second strategy is a push for redefining the roles of researcher, clinician and developer alike as morally responsible not only for ensuring that adequate protection and safeguards are in place but also for conveying their importance to the public in accessible ways^105^, ^106^, ^111^ while taking into account that public understandings of cybersecurity^84^ and attitudes towards data sharing are diverse.^83^, ^85^ Public engagement in professional education is another important future avenue towards improving the safety and acceptability of new AI‐assisted practices.^63^, ^164^, ^165^

### 4.3 Public engagement in the planning, development, implementation, evaluation and diffusion of AI technologies

#### 4.3.1 New contexts and opportunities for PPI

There is strong preliminary evidence that data‐intensive technologies can enhance PPI in research and caregiving. For example, public engagement in clinical research is being facilitated by the rapid transition to Internet‐based trials.^166^ Multiple studies reported that data collection and analysis techniques could enable patients to take an active role in their care through self‐monitoring,^62^, ^78^, ^84^, ^152^, ^167^ to contribute experiential insights into service‐orientated research,^63^ to maintain motivation and adherence to treatment,^97^, ^168^ or to facilitate engagement with clinicians, other patients, or friends and family.^59^, ^72^, ^106^ The development of creative digital approaches includes the following: online communities, digitization and redesign of psychology interventions, biometrics and data‐driven approaches, creative sharing of stories, symbolic engagements, and creative offerings of comfort and encouragements of self‐care.^169^

As such, data‐intensive technologies offer new possibilities for relationship‐based involvement (eg based on a patient's support network), especially for people with severe psychiatric disorders where good outcomes often entail the involvement of informal caregivers.^75^, ^118^ However, in the clinical context, carers may feel separated and distanced from the technical aspects of data gathering and analysis.

Data‐intensive technologies could break new ground in PPI for service design and quality improvement, for example as media for relaying real‐time feedback on service quality^77^ and patient‐reported outcomes (PRO),^170^ or for engaging underserved populations who are less likely to engage with specific services for sustained periods of time.^73^, ^93^, ^146^

Co‐design of virtual reality (VR) scenarios with young people shows promise,^171^ but little is known about the safety of implementing immersive VR technologies in sensitive settings.^172^ It is known that patient engagement in such technologies is negatively affected by poorly designed features, bugs and didactic information giving.^104^, ^173^ Other needs depend upon the type of user, for example those with severe insomnia^101^ or people who are homeless,^174^ and the practical/annoyance issues of using technologies. Wearable technologies tend to address a specific use case/health area, such as bedwetting^175^; however, this could result in non‐scalable and 'silo' solutions.^176^

Despite multiple examples of co‐design of mental health technologies (mostly apps or Web‐based information) with children and young people,^67^, ^177^, ^178^, ^179^, ^180^ future work will need to consider diversity in the user group, for example children experiencing psychosis.^181^

#### 4.3.2 Public awareness, preference and choice

The sheer diversity of data‐driven solutions to mental health drastically expands the available choices patients and the public face about whether to engage with such technologies or become involved in their design and implementation. Digital technologies could make it easier for some patients and broader publics to be involved in self‐care, caregiving, research and service design remotely and at flexible times. At the same time, other people might find themselves, or choose to be part of, a new minority of ‘others’ across a digital divide.

In one study, user design and testing of a Web‐based portal for dementia showed users felt an increased sense of autonomy and found the portal to be user‐friendly, helpful and efficient but felt that more information should be accessible.^182^ Moreover, data can be collected passively or through active user engagement (eg automatically tracked UV exposure vs. user‐inputted data on daily activities); various options can appeal for some patients and not others.^62^, ^183^ Clinicians prefer some technologies as opposed to others,^59^, ^72^, ^78^, ^103^ depending on features, functionalities or approaches. This has implications for involvement choices as some patients could be more likely to get involved in design of game‐based approaches,^184^ real‐world stories or problem‐solving tasks.^73^ Nevertheless, technologies can and should be adaptable to variability in individual preferences.^65^, ^66^, ^185^ and cultural contexts of use.^73^, ^104^, ^186^

#### 4.3.3 Patient and public trust

Patient trust is a major concern throughout the literature,^187^ not least because negative past experiences of care and fears of self‐disclosure and stigma have been known to fray service users' trust in mental health‐care providers.^59^, ^95^ Interestingly, chatbots and anonymous digital reporting have been found to increase patients' willingness to disclose sensitive information about their mental health.^77^, ^95^ Patients have even suggested that professional training makes greater use of virtual reality spaces to see things from the patient's perspective.^188^

Other studies caution that data‐intensive technologies may complicate relationships and information sharing.^26^, ^152^, ^189^ The health‐care provider, be they a person, avatar or computer program, requires good interpersonal competencies to build a working relationship ‘bond’ with the client^190^ and to inspire feelings of fondness,^191^ privacy, security^83^, ^84^, ^85^, ^160^, ^192^ and responsiveness.^193^

## 5 DISCUSSION

**FIGURE 3:**
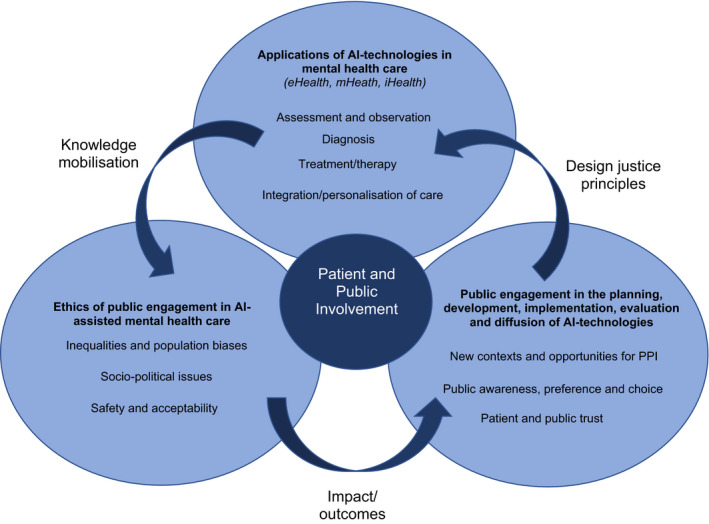
PPI in the conception and transition to AI‐assisted mental health care

AI technologies are being used to tackle many mental health challenges, including meeting service demand,^194^ supporting service improvement, improving access to clinicians, integrating care and support networks and eliciting feedback about services.^195^ A data‐rich AI‐assisted health environment holds great promise as a means to overcome enduring points of weakness in contemporary PPI initiatives, such that PPI can become more inclusive, wider reaching and accessible. Figure 3 illustrates the interrelationships between the themes of our results to provide an overview of an agenda for the future development of PPI in AI technologies for mental health described below.

### 5.1 Four principles for design justice in AI

#### 5.1.1 Meaningful and authentic public engagement in all areas of AI technologies can be supported and guided by the core principle that such technologies should sustain, heal, connect and empower people and communities

Design of AI technologies for mental health can be widened from a focus on addressing a problem of service demand, towards improving quality and safety, and protecting holistic well‐being from the perspective of diverse patients and healthy people. By developing inclusion frameworks that engage patients and healthy people, innovative AI technologies for mental health can be centred on the voices of those who are directly affected by the outcomes of the design process.^17^, ^196^, ^197^, ^198^ Practically or figuratively, putting digital intelligence into the hands of the person could enable them to take more control of their own health and well‐being. There is a need to encourage cultures of technology development that actively and ambitiously engage with people who are affected by the outcomes of such work—including groups of the public who stand to experience the worst effects or miss out on the benefits of technological innovations.

#### 5.1.2 Substantial ethical concerns, such as inequalities, cultural and population biases, safety, acceptability and broader socio‐political issues, could be better understood and moderated if design focused on the concerns of the community over the intentions of the designer

The case for public engagement is more complex in relation to those design processes that do not formally lie within the publicly funded sphere,^199^, ^200^ for example social media giants and technology designers who sell directly to the public. In practical terms, developing PPI in such territory means connecting communities of practice around issues, mobilizing the knowledge and expertise that exists in both public and private sectors, and finding avenues to incentivize and value PPI through existing regulatory, governance and public accountability structures.^90^

#### 5.1.3 AI technologies need to emerge from an accountable, accessible and collaborative process that describes how patients and the public have been involved

The literature reviewed here supports a call to approach the design of data‐intensive technologies as an ethical and political issue, as opposed to strictly technical.^6^, ^7^, ^10^, ^13^, ^201^, ^202^ There is a need to examine and challenge current power asymmetries (access to funding and networking, for example) in processes of designing AI technologies that will affect patients and the public, not just in the use or application of those technologies. PPI should play a central role in addressing the relative lack of guidelines for design and best practice in AI‐assisted care^56^, ^85^, ^107^, ^117^, ^160^ if data‐rich care tools are to be trusted as both effective and fair.^26^, ^111^, ^116^, ^161^

#### 5.1.4 AI developers and their designs can be informed by, and contribute to, shared knowledge in design and tools to support design justice

This is particularly important in relation to building shared knowledge about new contexts and opportunities for formal PPI, understanding the level of public awareness, preferences and choice, and tackling issues of trust and power/privilege between different actors in the system. Given that a lack of transparency about patient data remains a major concern for UK mental health activists,^80^ collective data ownership arrangements^203^ and open dialogues about the role of patient and public work in the production and interpretation of data^21^, ^170^ could help to build trust and willingness to engage in the design of AI‐assisted mental health care.

These principles offer some needed guidance about the public's role in imagining new uses of AI and creating the types of technologies that patients and the public need and want. PPI offers some way to counter the hegemony of commercial applications of AI and to create a more inclusive future through collaborative design. Further research is needed to explore how collaborative design can embed diverse values and socio‐cultural conceptions of human behaviour into digital machines. We have not addressed the issue of how appropriate inclusion frameworks can be developed at different levels of technology decision making and design or how commitment to these principles can be secured. The headline message of the UK 2019 State of the Nation Survey on accelerating AI in health and care was ‘Ground AI in ‘problems’ as expressed by the users of the health system’. From our analysis, we argue that this message needs to now include the fact that in some cases, the problem might be the AI.

## 6 CONCLUSION

The new data‐rich digital era creates multiple ethical issues and opportunities for public engagement in health and for development of formalized PPI in the design of AI‐assisted health care. Within the various bodies of research and innovation we have examined, we have found that patients and clinicians are generally in favour of data‐driven approaches to mental health as mechanisms for producing and mobilizing knowledge on the lived experience of patients outside clinical settings. Further research is needed to understand modes of public engagement in the different socio‐political, clinical and lived contexts of use of AI technologies, to examine the ethics of AI‐assisted health care and to develop new methods of PPI at every stage, from concept design to the final review of technologies in practice. PPI is a necessity for aligning advances in AI with egalitarian goals of equality, diversity and inclusion.

## CONFLICT OF INTEREST

There are no conflicts of interest.

## Data Availability

Data sharing is not applicable as no new data were generated.

## APPENDIX 1

Summary of all of the included articles in the review

**Table:** Note

Reference	Type of article	Context	Form of engagement	Relevant findings	Country
Achilles 2020	Review: intervention development and research design	Young people's adherence to e‐mental health	Review of the adherence literature, focusing on factors associated with improving adherence to e‐mental health among youth (no direct public engagement)	Key elements to improve adherence include: developing and communicating adherence guidelines based on individuals’ needs and symptom severity, including customizable features to provide a tailored experience and promote a sense of agency, including engagement checks and adopting a user‐centred approach by utilizing strategies such as co‐design.	Australia
Aji 2019	Research: Mixed‐methods study	Mobile apps for sleep disturbance	People with poor sleep and insomnia participated in qualitative (2 focus groups n = 9 and app reviews) and quantitative (online survey) approaches. (N = 9). An online survey tested themes identified from the focus groups against a larger population (N = 167). In addition, 434 user reviews of 6 mobile apps available on app stores.	This co‐design process involving end users through 3 methods consistently highlighted sleep tracking (through a diary and WD), alarms, and personalization as vital for engagement, although their implementation was commonly criticized in review. Engagement is negatively affected by poorly designed features, bugs, and didactic information which must be addressed. Other needs depend upon the type of user, for example, those with severe insomnia.	Australia
Alhuwail 2020	Review: systematic assessment of apps for Arabic speakers	Mobile apps for depression and anxiety for Arabic speakers	Review of 23 depression and anxiety apps, available free of charge to Arabic speakers. (no direct public engagement)	The results of this study clearly highlight the current gaps to address the needs of Arabic speakers‐ 16 apps provided general information about anxiety or depression, others focused on spirituality or herbal medicine. There is a need to involve expert health‐care professionals in the development of mental health apps and for health‐care providers to empower patients through discussing apps that are useful and discern them from those that can potentially cause harm.	Kuwait
Allan 2019	Research: Qualitative research	Monitoring early signs of psychosis relapse	149 mental health professionals, carers, and people diagnosed with psychosis participated in 25 focus groups in both Australia and the United Kingdom. An interview schedule informed by the normalization process theory was used to explore stakeholders' expectations about the implementation of the EMPOWER intervention.	A hypothetical implementation framework synthesized from stakeholder implementation expectations provides an opportunity to compare actual implementation data gathered during an on‐going clinical trial, giving valuable insights into the accuracy of these stakeholders' previous expectations. This is among the first studies to assess and record implementation expectations for a newly developed digital intervention for psychosis in advance of testing in a clinical trial.	UK/Australia
Baghaei 2019	Research: Design and initial proof of concept	Early intervention to improve resilience and well‐being in young people	Co‐design virtual reality scenarios with young people, which focuses on real‐world situations that impact the young people the most and assists them to view these experiences with a self‐compassionate lens.	Co‐design of virtual reality scenarios with young people, which focuses on real‐world situations that impact the sample group most and assists them to view these experiences with a self‐compassionate lens. This is achieved by being taught compassionate manners of responding to a scenario and by switching perspective.	
Baig 2017	Research: Review	Wearable patient monitoring systems	Review of barriers and challenges of wearable patient monitoring (WPM) solutions adopted by clinicians in acute, as well as in community, care settings.	A review of WPM systems using advanced sensors, wearable technology, and secure and effective communication platforms between clinicians and patients. A total of 791 articles were screened and 20 were selected for this review. Most studies focused on the system aspects of WPM solutions including advanced sensors, wireless data collection, communication platform and clinical usability based on a specific area or disease. The current studies are progressing with localized sensor‐software integration to solve a specific use case/health area using non‐scalable and 'silo' solutions. There is further work required regarding interoperability and clinical acceptance challenges.	New Zealand
Bauer 2015	Research: Validation of software	Self‐reporting software for bipolar disorder	Self‐reported mood ratings on ChronoRecord and clinician ratings on the YMRS were obtained on the same day from 27 inpatients and 80 outpatients.	ChronoRecord software was developed to automate daily self‐reporting by patients with bipolar disorder. Validation of ChronoRecord by patients with mania shows the self‐reporting is a valid way of monitoring bipolar disorder.	Germany
Bedi 2015	Research: Automated analysis of free speech	Young people at high risk of psychosis	Thirty‐four CHR youths (11 females) had baseline interviews and were assessed quarterly for up to 2.5 y; five transitioned to psychosis. Using automated analysis, transcripts of interviews were evaluated for semantic and syntactic features predicting later psychosis onset.	Findings support the utility of automated speech analysis to measure subtle, clinically relevant mental state changes in emergent psychosis. Recent developments in computer science, including natural language processing, could provide the foundation for future development of objective clinical tests for psychiatry.	USA
Ben‐Zeev 2016	Research: Evaluation	mHealth for schizophrenia	Participants with schizophrenia spectrum disorders (n = 342) used an mHealth intervention for 6 mo after hospitalization	The findings demonstrated that individuals with schizophrenia spectrum disorders can actively engage with a clinically supported mobile phone intervention for up to 6 mo following hospital discharge. mHealth may be useful in reaching a clinical population that is typically difficult to engage during high‐risk periods.	USA
Bergin 2020	Review: scoping review of the design and reporting of research	Preventative digital health interventions (DHIs) for children and young people	Review of co‐design studies (no direct public engagement)	The review found limited reporting of user co‐design involvement (2 out of 21 interventions) in the design of DHIs. Argues that co‐design processes with children and young people need to be reported and recognized as an important factor in design.	UK
Berrouiguet 2018	Research: Review	Participatory and personalized medicine in mental health	Review of intelligent health in mental health	Intelligent health (iHealth) further builds on and expands eHealth by adding novel built‐in data analysis approaches based on (1) incorporation of new technologies into clinical practice to enhance real‐time self‐monitoring, (2) extension of assessment to the patient's environment including caregivers, and (3) data processing using data mining to support medical decision making and personalized medicine.	France
Bevan Jones 2020	Research: Review	Digital mental health technologies for children and young people	Review of the literature and practice in the co‐design of digital mental health technologies with children and young people.	The review identified 25 original articles and 30 digital mental health technologies that were designed/developed with children and young people. The themes identified were: principles of co‐design (including potential stakeholders and stages of involvement), methods of involving and engaging the range of users, co‐designing the prototype and the challenges of co‐design.	UK
Blanco 2019	Research: Conceptual development	Social‐based patient support	Design and evaluation of a social‐based solution for patient support	This work contributes with the design, development and assessment of a new concept: Micro ad hoc Health Social Networks (uHSN), in order to create a social‐based solution for the support of patients with chronic diseases. This new paradigm of uHSN defines two interaction areas (‘backstage’ and ‘onstage’) and a transversal concept of ‘network spaces segments’ to provide timely interaction among all the involved profiles and guaranteeing qualitative relationships.	Spain
Boydell 2017	Research: Participatory inquiry	Social inclusion of young people experiencing psychosis	Participatory inquiry involved young people sharing their experiences of seeking help. digital story telling (DST) is emerging as an arts‐based participatory research method to enhance understanding of ‘lived experience’, promote social connections and address power imbalances.	Digital stories are a powerful medium for young people experiencing psychosis to tell stories about issues they identify as important.	Australia
Brice 2015	Research: Review	Online clinical trials	Review of online trials to determine how many trials have used internet‐based technologies; how they have been used; and how use has developed over time to build an open access resource to support a public‐led research agenda (no direct public engagement)	The review found 1992 internet‐based trials to March 2015. The number of reported studies increased substantially over the study timeframe. The largest number of trials were conducted in the USA (49.7%), followed by The Netherlands (10.2%); Australia (8.5%); the United Kingdom (5.8%); Sweden (4.6%); Canada (4%); and Germany (2.6%). There is a predominance of interventions addressing core public health challenges including obesity (8.6%), smoking cessation (5.9%), alcohol abuse (7.7%) and physical activity (10.2%); in mental health issues such as depression (10.9%) and anxiety (5.6%); and conditions where self‐management (16.6%) or monitoring (8.1%) is a major feature of care.	UK
Brown 2019	Research: review	Digital post‐traumatic support	Review of current approaches to supporting mental well‐being outside of therapy	This article offers a reflection on possibilities for co‐design in a digital economy. Six themes of creative approaches from 83 existing interventions: online communities, digitization and redesign of psychology intervention, biometrics and data‐driven, creative sharing of stories, symbolic engagements, and creative offerings of comfort and encouragements of self‐care. Involvement of trusted others in the co‐design of care entanglements is proposed, suggesting that these involvements would allow for reciprocal care encounters to be personalized towards those who have experienced trauma and their trusted others.	Australia
Bruns 2016	Design: System development	Care coordination for people with complex needs	User/stakeholder perspectives were captured at multiple points and resulted in an electronic behavioural health information system (EBHIS) specific to the wraparound care coordination model for youth with serious emotional and behavioural disorders.	A series of six small studies informed system development across four phases of effort‐predevelopment, development, initial user testing, and commercialization. The research team's experiences reinforce the opportunity presented by EBHIS to improve care coordination for populations with complex needs, while also pointing to a litany of barriers and challenges to be overcome to implement such technologies.	USA
Bucci 2018	Research: Co‐design of app	Early signs of distress in psychosis	Co‐design with patients and stakeholders Actissist is a theory‐informed smartphone app targeting distress in early psychosis.	The success of digital health interventions is not merely determined by patient uptake it will ultimately be determined by patients and staff, both of whom are key end‐users. Mental health professionals and patients often express concerns about data security, safety and risk information being robustly handled.	UK
Bucci 2019	Research: Review	Digital health technology in mental health	Reviews of digital health technology assessment and intervention primarily in secondary service mental health care, including the barriers and facilitators to adopting and implementing digitally mediated interventions in service delivery.	These developments point to a need for both theory‐ and data‐driven approaches to digital health care. More advanced methodologies are needed to keep up with the pace of digital technology development. The need for co‐production of digital tools with and for people with chronic and mental health difficulties, and implications of digital technology for psychotherapy practice, will be central to this development.	UK
Buitenweg 2019	Research: co‐creative development	Personalized quality of life assessment app for people with severe mental health problems	3 groups of individuals with severe mental health problems: (1) people with psychiatric problems, (2) people treated in forensic psychiatry, and (3) people who are homeless. A group of 59 participants contributed to the 6 iterations of the co‐creative development of the QoL‐ME.	In the brainstorming stage, participants stressed the importance of privacy and data security and of receiving feedback when answering questionnaires. Participants in the design stage indicated a preference for paging over scrolling, linear navigation, a clean and minimalist layout, the use of touchscreen functionality in various modes of interaction, and the use of visual analogue scales. The usability evaluation in the usability stage revealed good to excellent usability. The co‐creative development of the QoL‐ME resulted in an app that corresponds to the preferences of participants and has strong usability. Further research is needed to evaluate the psychometric quality of the QoL‐ME and to investigate its usefulness in practice.	The Netherlands
Caswell 2020	Research: Product development and testing	Product development for bedwetting (MyPAD)	A PPI workshop in the form of a focus group, made up of children and their parents, was conducted during the early stage of the MyPAD product development. The key research questions were: What were the families’ experiences of using existing post‐void enuresis alarms? What do families like about the MyPAD prototype? and What do families not like about the MyPAD prototype?	The qualitative data derived from the focus group discussion was incredibly valuable as it enabled the research and design team to experience the perspectives of the families in terms of the challenges and conflicts of managing the condition and the limited utility of existing post‐void alarms. This has improved the researcher's understanding of the social and environmental challenges that need to be considered during the design process.	UK
Chancellor 2019	Research: Review	Inferring mental health states from social media	Review and taxonomy of concerns and ethical challenges	Identifies three areas of tension: ethics committees and the gap of social media research; questions of validity, data, and machine learning; and implications of this research for key stakeholders.	USA
Cho 2019	Research: Literature review	Machine learning	Review of mental illness diagnostic using ML techniques (no direct public engagement)	This paper reviews the research of diagnosing mental illness using ML algorithm and suggests how ML techniques can be employed and worked in practice.	Canada
Christie 2019	Research: design of digital intervention	Gamifying CBT to deliver emotional health treatment to young people on smartphones	Focus groups and interactive workshops with New Zealand, Māori and Pasifika youth were used to inform the co‐design process. Clinicians worked iteratively with experienced game designers to co‐create a youth CBT digital intervention.	Gamifying CBT involves selecting suitable interventions, adapting them to a digital format while applying gamification principles. The Quest modular app is set on an ocean and the user travels between islands to learn six evidence‐based skills. These include a relaxation/mindfulness activity, activity planning, a gratitude journal plus problem‐solving and communication skills training. Gamified features aim to increase user engagement.	New Zealand
Cirillo 2020	Review: biomedical data types and AI technologies	Sex and gender gaps in a subset of biomedical technologies used in precision medicine	Review of AI technologies (no direct public engagement)	Most of the currently used biomedical AI technologies do not account for bias detection. The design of the majority of algorithms ignore the sex and gender dimension and its contribution to health and disease differences among individuals. Failure in accounting for these differences will generate sub‐optimal results and produce mistakes as well as discriminatory outcomes.	Spain
Cohen 2020	Research: counselling service research	Mobile technologies for stress management	Counselling centre clinicians and students at two large Midwestern universities. Clinicians completed online questionnaires on their current practices and interest in digital mental health tools. Students and clinicians completed co‐design workshops. In subsequent individual interviews, students identified barriers to care.	Students and clinicians recognize barriers to face‐to‐face therapy and are eager to collaborate to identify opportunities to address barriers to mental health services using digital mental health tools as a potential opportunity for support. The authors describe the benefits of including students in discussions of treatment resources.	USA
Conlon 2020	Research: Mixed‐methods descriptive study	Perceptions of realism in digital scenarios for mental health nurse education	A co‐productive active learning approach explored perceptions of authenticity using an authenticity scale. Stakeholder facilitated workshop discussions explored perceptions of the educational scenarios. Participants included mental health nursing students, people with lived experience and family carers. A mixed‐methods descriptive study using the adapted authenticity scale to rate four exemplar scenarios along with thematic analysis of workshop discussions.	Digital audio compared less well to visual media in authenticity scales. Still photobook‐style images were also perceived as less authentic than dramatic film employing professional actors. Digital media must be selected carefully not just in relation to the education needs of the student but in relation to their social, cultural norms and digital skills. Creating digital scenarios co‐productively could contribute to a teaching resource that holds authenticity and real‐world relevance.	UK
Conlon 2020	Research: Qualitative exploration of the suitability of digital stories as pedagogical tools	Digital storytelling as an approach designed to apply the theory of authentic learning in a co‐productive context	A participant group (n = 7) comprising family carers, people with lived experience and mental health nursing students were invited to join two facilitated workshops. The group reviewed four contrasting forms of digital stories with the aim of eliciting and sharing their perspectives.	Digital audio compared less well to visual media in authenticity scales. Still photobook‐style images were also perceived to be less authentic than dramatic film employing professional actors. Furthermore, it was found that the essence of authenticity became richer as the process and activities of co‐productive engagement developed. It is proposed that creating digital scenarios co‐productively provides a relational environment in which the essence of authenticity can be felt and expressed.	UK
Craven 2019	Research: Co‐design method	Design of a visualization toolbox for people to track their mental health	A participant co‐design methodology, Double Diamond from the Design Council (Great Britain), was used. Two design workshops were held with 13 and 12 experts by experience involved, respectively, including two peer researchers (co‐authors) and two individual‐carer dyads in each workshop, with over 50% of those being present in both workshops.	While participants concurred on a range of typical dimensions of well‐being, the individual visualizations generated were in contrast to the techniques currently employed by existing digital well‐being apps and there was a great diversity in preference for different visualization types. Participants considered personal visualizations to be useful as self‐administered interventions or as a step towards seeking help, as well as being tools for self‐appraisal.	UK
Crosby 2020	Research: Qualitative research	Self‐care for depression and anxiety	Interview study of 14 individuals living in England with a diagnosis of depression or an anxiety disorder, who used smartphone apps as part of self‐care.	People engage with apps in a straightforward and uncomplicated manner, leading to immediate symptomatic alleviation, but to limited longer term benefit. The contradiction between the apps’ promise as tools of individual empowerment, with their ability to promote responsibilizing frameworks that restrain users’ reflexivity, is central to their implications. Apps can thus contribute to isolation from interpersonal support and promote reductionist biomedical conceptualizations of mental ill health.	UK
Davies 2019	Research: design development	Online support for family caregivers of people with dementia at the end of life	Adopting an iterative approach and co‐production methods the development process consisted of four stages including a research development group, developing the intervention prototype and interviews with caregivers testing the prototype website.	Involvement of caregivers using qualitative interviews identified four targets for the intervention for family caregivers of people with dementia: 1) feeling prepared and equipped; 2) feeling connected and supported; 3) valuing themselves as a caregiver and as an individual; 4) maintaining control of the caring situation and being the coordinator of care.	UK
Davis 2020	Research: pharmacoepidemiology research	Delivering evidence about drug safety and effectiveness in mental health	The abundance of digital data has facilitated pharmacoepidemiology and, in particular, observational research on the effectiveness of real‐world medication.	With developments in data resources and analytical techniques, guidelines are beginning to include evidence from robust observational pharmacoepidemiological studies alongside randomized controlled trials. Collaboration between guideline writers and researchers involved in pharmacoepidemiology could help researchers to answer the questions that are important to policy makers and ensure that results are integrated into the evidence base. Further development of statistical and data science techniques, alongside public engagement and capacity building (data resources and researcher base), will be necessary to take full advantage of future opportunities.	UK
Dewa 2019	Research: qualitative study	Young adults’ perceptions of using wearables, social media and other technologies to detect worsening mental health	The study was co‐produced with 16 young adults with past mental health difficulties. Semi‐structured interviews were conducted with young adults with a severe mental health condition in a private room at a community mental health site. Data was triangulated by comparing codes and ideas across the two co‐researchers and two researchers over two virtual meetings. Themes were finalized and presented in a thematic map.	Involvement of young adults revealed four main themes: dealing with mental health symptoms, signs of mental health deterioration, technology concerns and technological applications to identify worsening mental health. Wearables and mobile apps were considered acceptable and feasible to detect mental health deterioration in real‐time if they could measure changes in sleep patterns, mood or activity levels as signs of deterioration. Getting help earlier was deemed essential particularly in reference to dissatisfaction with the current non‐technological mental health services. However, patients identified issues to consider before implementation including practicality, safeguarding and patient preference. Wearables and mobile apps could be viable technological options to help detect deterioration in young people in order to intervene early and avoid delay in accessing mental health services. However, immediate action following detection is required for the patient to trust and use the intervention.	UK
Dodd 2017	Research: Qualitative interviews	Online intervention for bipolar disorder	Participants from a research trial who had been allocated to receive ERPonline were purposively sampled (n = 19) to participate in telephone‐based, in‐depth qualitative interviews about their experiences.	Participants took part due to the convenient, flexible and rewarding aspects of the trial design, as well as a desire to improve the mental health of themselves and others. Barriers included extensive assessments, practical difficulties and mood. ERPonline was generally considered to be accessible, relevant and straightforward, but there were individual preferences regarding design, content and who it was for. Several participants reported positive changes, but there was a sense that digital interventions should not replace routine care. There are a number of barriers and facilitators to consider when evaluating and implementing digital interventions. Individual preferences and human contact were key factors for both trial design and engagement with an online intervention.	UK
Doukani 2020	Research: qualitative study	Development of a conceptual framework of a cognitive behavioural therapy intervention for depression in primary mental health care	Patient involvement was enlisted to collaboratively shape the design of the project from the onset, before data collection. In‐depth semi‐structured interviews were carried out with participants who experienced b‐CBT as part of the E‐compared trial	Qualitative interviews that were guided by theory and patient involvement, revealed four themes: (1) a health‐care provider (PWP and computerized programme) with good interpersonal competencies for building a working relationship with the client (‘bond’); (2) collaborative efforts between the client and the provider to appropriately identify what the client hopes to achieve through therapy (‘goals’); (3) the selection of acceptable therapeutic activities that address client goals and the availability of responsive support (‘task’) and (4) the promotion of active engagement and autonomous problem‐solving (‘usability heuristics’).	UK
Easton 2019	Research: Co‐design and acceptability testing	Physical and mental comorbidities	Co‐design of a virtual agent with individuals living with physical and mental comorbidities	Patients did not explicitly separate mental and physical health needs, although the content they developed for the virtual agent had a clear psychological approach. Supported self‐management delivered via an autonomous virtual agent was acceptable to the participants. A co‐design process has allowed the research team to identify key design principles, content, and functionality to underpin an autonomous agent for delivering self‐management support to older adults living with COPD and potentially other LTCs.	UK
Fairburn 2015	Research: systematic clinical appraisal	Apps for eating disorders	A search of the major app stores identified 39 were primarily designed for people with eating disorders and 5 for professionals (no direct public engagement)	The clinical utility of the existing apps is not clear or fully utilized. Some are capable of tracking key features over time, but none has the functions required for analytic self‐monitoring as in cognitive behavioural treatments.	UK
Farina 2019	Research: Measuring patient activity	Monitoring activity in people with dementia	26 community‐dwelling, people with mild dementia were asked to wear an activity monitor (GENEactiv Original) over a 1‐mo period.	Asking people with dementia to wear a wrist‐worn activity monitor for prolonged periods appears to be both feasible and acceptable. Researchers need to consider the needs and preferences of the sample population prior to selecting activity monitors.	UK
Firth 2017	Research: meta‐analysis of randomized controlled trials	The efficacy of smartphone‐based mental health interventions for depressive symptoms	Systematic review and meta‐analysis of all randomized clinical trials (RCTs) reporting the effects of psychological interventions delivered via smartphone on symptoms of anxiety (no direct public engagement)	This meta‐analysis shows that psychological interventions delivered via smartphone devices can reduce anxiety. Future research should aim to develop pragmatic methods for implementing smartphone‐based support for people with anxiety, while also comparing the efficacy of these interventions to standard face‐to‐face psychological care.	Australia
Fleming 2019	Research: scoping processes	Digital therapy for adolescent mental health	Interactive workshops and focus groups were held with young people. Participants (N = 58) engaged in 2 whānau (extended family) focus groups (n = 4 and n = 5), 2 school‐ or community‐based focus groups (n = 9 each), and 2 workshops (n = 11 and n = 20).	The authors identified 3 overarching themes: (1) Digital mental health tools are unlikely to be successful if they rely solely on youth help‐seeking. (2) A single approach is unlikely to appeal to all. Participants had diverse, non‐compatible preferences in terms of look or feel of an app or digital tool. The authors identified 4 user groups players or gamers, engagers, sceptics, and straight‐talkers. These groups differed by age and degree of current mental health need and preferred gamified or fun approaches, were open to a range of approaches, were generally disinterested, or preferred direct‐to‐the‐point, serious approaches, respectively. (3) Digital mental health tools should provide an immediate response to a range of different issues and challenges that a young person may face. Defining the preferences of different groups of users may be important for increasing engagement with digital therapies even within specific population and mental health–need groups. This study demonstrates the importance of scoping possible user needs to inform design processes.	New Zealand
Flemming 2018	Research: Systematic review	Digital self‐help interventions	Systematic review of studies reporting user uptake and/or usage data from implemented digital self‐help interventions for the treatment or prevention of depression or anxiety, or the enhancement of mood	Available data suggest that uptake and engagement vary widely among the handful of implemented digital self‐help apps and programs that have reported this, and that usage may vary from that reported in trials. Implementation data should be routinely gathered and reported to facilitate improved uptake and engagement.	New Zealand
Gajecki 2014	Research: randomized controlled study	Mobile phone brief intervention applications for risky alcohol use among university students:	Students at 2 universities (1932) were involved in testing an app (Promillekoll) a web‐based app (PartyPlanner), developed by the research group	Smartphone apps can make brief interventions available to large numbers of university students however they did not appear to affect alcohol consumption and one app may have led to a negative effect among men. Future research should: (1) explore ways to increase user retention, (2) include apps facilitating technical manipulation for evaluation of added components, (3) explore the effects of adapting app content to possible gender differences, and (4) offer additional interventions to high‐risk users.	Sweden
Gault 2019	Co‐production for service improvement: Developing a	Training for mental health professionals to enhance medication adherence	To co‐produce consensus on the key issues important in educating mental health‐care professionals to optimize mental health medication adherence in Black, Asian and Minority Ethnic (BAME) groups.	Service user and carer participants' perspectives substantially altered the original research design. The need to educate students rather than trained professionals was emphasized, and they suggested that educational content should be packaged in a contemporary manner (a virtual reality experience). Findings indicated that education should focus upon understanding the impact of taking prescribed antipsychotic medication on both service users and carers.	UK
Gindidis 2019	Research: systematic scoping review	Adolescent mental health treatment using mobile apps	Review of mental health apps	Function‐based categorization of apps permits practitioners to consider and select apps that ‘fit’ best with individualized, evidence‐based practice approaches.	Australia
Gonsalves 2019	Research: design and development	Design of a smartphone game for adolescent mental health	Person‐centred approach including focus group discussions with (n = 46) student participants and co‐design workshops with (n = 22) student participants and user testing with (n = 50) student participants. Participants were aged 12‐17 y and recruited from local schools in New Delhi and Goa, including a subgroup with self‐identified mental health needs (n = 6).	Formative data from existing primary sources, new focus groups and co‐design workshops supported a blended format for delivering a brief problem‐solving intervention. User testing with prototypes identified a need for simplification of language, use of concrete examples of concepts and practice elements to enhance engagement. There were also indications that participants most valued relatability and interactivity within real‐world stories with judicious support from an in‐app guide. The final prototype comprised a set of interactive and gamified vignettes and a structured set of problem‐solving questions to consolidate and generalize learning while encouraging real‐world application.	India
Goodyear‐Smith 2018	Research:	Screening for risky behaviour and mental health in young people	Co‐design participatory research to assess acceptability and feasibility of YouthCHAT with successive roll‐out to clinics.	Opportunistic screening for mental health concerns and other risky health behaviours during adolescence can yield significant health gains and prevent unnecessary morbidity and mortality. The systematic approaches to screening and provision of algorithms for stepped‐care intervention will assist in delivering time efficient, early, more comprehensive interventions for youth with mental health concerns and other health compromising behaviours. The early detection of concerns and facilitation to evidence‐based interventions has the potential to lead to improved health outcomes, particularly for underserved indigenous populations.	New Zealand
Graham 2019	Discussion drawing on research studies	AI in predicting, diagnosing and classifying neurocognitive impairments	Discussion paper providing a conceptual overview of AI application (no direct public engagement)	This paper serves to acquaint clinicians and other stakeholders with the use, benefits, and limitations of AI for predicting, diagnosing, and classifying mild and major neurocognitive impairments, by providing a conceptual overview of this topic with emphasis on the features explored and AI techniques employed. The authors present studies that fell into six categories of features used for these purposes: (1) sociodemographics; (2) clinical and psychometric assessments; (3) neuroimaging and neurophysiology; (4) electronic health records and claims; (5) novel assessments (eg sensors for digital data); and (6) genomics/other omics. For each category they provide examples of AI approaches, including supervised and unsupervised ML, deep learning, and natural language processing. They conclude that AI technology, still nascent in health care, has great potential to transform the way we diagnose and treat patients with neurocognitive disorders.	USA
Gumley 2020	Research: protocol for a feasibility cluster randomized controlled trial	Early signs monitoring to prevent relapse in psychosis and promote well‐being, engagement, and recovery	Patient and carer engagement in a development of a mobile phone App blended with peer support	EMPOWER is a mobile phone app that enables the monitoring of well‐being and possible early warning signs of relapse on a daily basis. Patients and carers will be involved in this feasibility study to inform recruitment and retention to the trial and the acceptability, usability, safety, and outcome signals of the EMPOWER intervention.	UK
Hackett 2018	Research: Experience‐based co‐design	Improving quality in youth mental health services	Experience‐based co‐design (EBCD), involving young people aged 16‐24 with a mental disorder (n = 19), identified caregivers (n = 12) and service providers (n = 14). Experience data were collected using multiple approaches including interviews, a suite of online and smartphone applications (n = 22), and a co‐design event (n = 16) and analysed to extract touch points. These touch points were used to prioritize and co‐design a user‐driven prototype of a questionnaire to provide feedback to service providers.	By using EBCD to capture in‐depth data regarding experiences of young people, their caregivers and service providers, study participants have begun to establish a baseline for acceptable quality of mental health care for young people.	Canada
Hensel 2019	Research: Review and case study	Digital health solutions for Indigenous mental health	Review of digital health solutions being used for Indigenous mental well‐being and local experience with a rural telemental health service for Indigenous youth	The review finds that while the use of digital health solutions for Indigenous mental well‐being holds promise, there is a limited evidence base for most of them. Future efforts to expand the use of digital solutions in this population should adhere to best practices for the delivery of Indigenous health services.	Canada
Henshall 2019	Research: Focus group study	Digital clinical decision support tool (DST)	Qualitative study at a mental health and community hospital involving four patients/carers and four psychiatrists were recruited to two focus groups to explore their perceptions of the tool.	Findings demonstrated a high degree of acceptability and potential usability of the DST for patients and psychiatrists. The main themes to emerge relating to the DST were ‘prescribing preferences and practices’, ‘consideration and awareness of side effects’, ‘app content, layout and accessibility’, ‘influence on clinical practice’ and ‘role in decision‐making’.	UK
Hodson 2019	Research:	Crisis planning for young people	Community‐based project to collaboratively research, design and test an integrated digital crisis planning tool for youth ages 13‐24. The project was established with several guiding principles: that students should act as the front line researchers and designers with the guidance of faculty and community partners; that we should aim to move beyond consultation to truly participatory design methods involving end users in needs identification, idea generation, design development and testing; and that by having the students interface with the end users, we would alter the power balance by building trust and avoiding a top‐down approach.	Giving the students responsibility for much of the interaction with the end users had risks. The seriousness of the project and its potential for real impact –positive or negative ‐inspired the students to perform at an even higher level. They identified with the issue of mental health, a high‐profile subject on campuses right now, and they wanted to use their design training to help others. Their dedication to the project grew stronger as they became accountable to real ‘clients’ who were even more invested in the outcome.	USA
Husebo 2020	Review: systematic review	Sensing technology to monitor behavioural and psychological symptoms and to assess treatment response in people with dementia	Review of studies of sensing technologies in dementia (no direct public engagement)	34 studies of (1) wearable sensors, (2) non‐wearable motion sensor technologies, and (3) assistive technologies/smart home technologies. Half of the studies investigated how temporarily dense data on motion can be utilized as a proxy for behaviour, such as sleep disturbances, agitation and wandering. Up to half of the studies represented proof of concept, acceptability and/or feasibility testing. Overall, the technology was regarded as non‐intrusive and well accepted. The authors highlight awareness of legal regulations, data risk assessment, and patient and public involvement, with a necessary framework for sustainable ethical innovation in health‐care technology. The success of this field will depend on interdisciplinary cooperation and the advance in sustainable ethic innovation.	Norway
Husky 2014	Research: Comparative study	Ecological Momentary Assessment	Four groups of people at varying risk for suicide completed an Ecological Momentary Assessment study by responding to five electronic assessments per day over a 1‐wk period. Samples included healthy controls (n = 13), affective controls (n = 21), past suicide attempters (n = 20), and recent suicide attempters (n = 42).	The findings provide support for the use of Ecological Momentary Assessment in the study of suicidal ideation and suggest that mobile technologies represent new opportunities for the assessment of high‐risk cognitive states experienced by patients in daily life.	France
Jacobson 2020	Research: review	Ethical guidelines for apps	Review of ethical dilemmas posed by mobile health and machine learning in psychiatry research (no direct public engagement)	Ethical dilemmas regarding researchers’ duties to: (i) monitor adverse events and intervene accordingly; (ii) obtain fully informed, voluntary consent; (iii) protect the privacy of participants; and (iv) increase the transparency of powerful, machine‐learning models to ensure they can be applied ethically and fairly in psychiatric care.	USA
Jones 2016	Research: review	Ethics of health apps	Review of ethical guidelines for mobile app development within health and mental health fields	Currently there are no ethical guidelines for mobile health (mHealth) applications (apps) despite the rapid innovation and use of mobile technologies in the health‐care field. Privacy and confidentiality are of primary concerns and app developers and providers can safeguard against violations of privacy and confidentiality.	USA
Jones 2020	Research: mixed‐methods (quantitative and qualitative) feasibility evaluation	Digital intervention for adolescent depression	Adolescents (n = 44) with or at elevated risk of depression and their parents and carers (n = 31) were recruited from mental health services, school counsellors and nurses, and participants from a previous study. 82% completed questionnaires before and after the programme related to the feasibility and acceptability of the programme and evaluation process, and changes in mood, knowledge, attitudes, and behaviour, and their Web usage was monitored. A subsample of 19 were interviewed	Key themes from the interviews and groups related to the design features, sections and content, and integration and context of the programme in the young person's life. Overall, the participants found the intervention engaging, clear, user‐friendly, and comprehensive, and stated that it could be integrated into existing services. Young people found the ‘Self‐help’ section and ‘Mood monitor’ particularly helpful. The findings provided initial support for the intervention programme theory, for example, depression literacy improved after using the intervention. Findings from this early stage evaluation suggest that MoodHwb and the assessment process were feasible and acceptable, and that the intervention has the potential to be helpful for young people, families and carers as an early intervention programme in health, education, social, and youth services and charities. The authors suggest a randomized controlled trial is needed to further evaluate the digital programme.	UK
Jones 2020	Review: Literature and practice in the co‐design of digital mental health technologies with children and young people	Co‐design of digital mental health technologies with children and young people	Review of 30 studies using co‐design (no direct public engagement)	The review found 30 digital mental health technologies that were designed/developed with children and young people. The themes identified were as follows: principles of co‐design (including potential stakeholders and stages of involvement), methods of involving and engaging the range of users, co‐designing the prototype and the challenges of co‐design. Co‐design involves all relevant stakeholders throughout the life and research cycle of the programme. The authors suggest future work in this field will need to consider the changing face of technology, methods of engaging with the diversity in the user group, and the evaluation of the co‐design process and its impact on the technology.	UK
Kidd 2019	Research: feasibility and outcomes study	Multi‐function mobile health for schizophrenia	App4Independence use metrics were assessed as was qualitative feedback through semi‐structured interview. Among the 38 individuals with a primary psychosis who participated, there was no research attrition and classic retention on the app was 52.5%.	This study contributes to the small but emergent body of work investigating digital health approaches in severe mental illness populations.	Canada
Kipping 2016	Research: Quantitative evaluation	Web‐based portal for service users	Mental health service users (n = 461) accessed personalized health information via a web‐based portal over a year	Users felt an increased sense of autonomy and found the portal to be user‐friendly, helpful, and efficient but felt that more information should be accessible.	Canada
Klein 2014	Research: Quantitative research	Computerized recovery support programme for addiction	Patients accessed individually tailored clinical content in a multimedia format over 18 mo following residential treatment.	Low engagement with computerized health programmes is a widespread problem. Several factors were found to predict programme engagement, including several demographic variables, the number of recovery coach contacts, motivation to be in recovery, and attendance at 12‐step groups.	USA
Knoll 2018	Research: Conceptual model development	Environments that support adolescent health and well‐being	An urban health model and conceptual framework for researching environments that support health and well‐being in 10‐19‐y olds	Based on a review of the evidence from urban planning and environmental psychology literature, this article emphasizes the need for a more adolescent‐responsive urban design process, the need for more research into age‐specific urban affordances; integration of new technologies to forge mobility in and engagement with in the co‐design of cities allowing stakeholders to make better‐informed planning decisions.	Germany
Kreyenbuhl 2019	Research: Development of an app and clinical interface	Adherence to medication	Informed by the information‐motivation‐behavioural (IMB) skills model and using the iterative process of user‐centred design, the researchers collaborated with individuals with schizophrenia and psychiatrists to develop an interactive smartphone application and web‐based clinician interface, MedActive, for improving adherence to oral antipsychotic treatment.	In a 2 wk open trial completed by 7 individuals with schizophrenia and their psychiatrists, MedActive was determined to be both feasible and acceptable, with patient participants responding to 80% of all scheduled EMAs and providing positive evaluations of their use of the application. Psychiatrist participants were interested in viewing the information provided on the MedActive clinician interface, but cited practical barriers to regularly accessing it and integrating into their daily practice.	USA
Kruczek 2019	Research: Review	e‐health in mental health care	Review of e‐health tools targeting vulnerable patient populations.	eHealth technologies support increased patient participation in health‐care decision making. This approach allows reducing costs and improving effectiveness of psychotherapy via, for example increasing treatment intensity using digital technologies, or helping patients to integrate therapeutic strategies in their daily life between sessions. However, there are some limitations: financial, language and literacy barriers, power supply issues, data security and privacy issues.	Poland
Kumar 2019	Discussion article and engagement framework	The role of mental health practitioners in levering technology	Theorises patients and users as key stakeholders in the development of technological advances in mental health care	The article calls for a more proactive role of the mental health practitioner (MHP) in driving change in terms of leveraging technology in mental health settings. It looks at how certain tools can be incorporated across a range of scenarios from wellness applications and facilitating medical adherence to aiding crisis intervention and extending quality care services in remotes areas. The article briefly outlines a framework involving various stakeholders at different levels as well as the channels in which the technology can be leveraged while keeping the patients' rights front and centre. The potential barriers that an ‘e‐ready’ MHP can expect and directions for moving ahead are discussed, keeping a critical eye on the lacunae in using technology.	India
Kuru 2020	Design research: Product development and testing	Intelligent autonomous treatment of bedwetting	Children and parents involved at multiple stages of product development and testing – from ‘proof of concept’, device development, intelligent software development, a user‐friendly smartphone application, bedside alarm box, and a dedicated undergarment, and self‐adhesive gel pad	Involving children and parents at multiple stages of the design process has helped to develop a useful and usable design. An enhanced device will be tested with children with NE at their homes for 14 wk, to gain feedback relating to wearability and data collection involving the cloud platform	UK
Larsen 2019	Research: Review of apps	Mental health self‐help apps	Google Play and iTunes were searched for apps related to depression, self‐harm, substance use, anxiety, and schizophrenia. (no direct public engagement)	Seventy‐three apps were coded, and the majority (64%) claimed effectiveness at diagnosing a mental health condition, or improving symptoms, mood or self‐management. Scientific language was most frequently used to support these effectiveness claims (44%), although this was not backed up by citations to research evidence.	Australia
Lawn 2019	Research: Study protocol	Smoking cessation for smokers with severe mental Illness (SMI)	Using co‐design principles, the researchers will adapt the Kick.it smartphone App in collaboration with a small sample of current and ex‐smokers with SMI.	This pilot work will inform a larger definitive trial. Dependent on recruitment success, the project may extend to also include smokers with SMI who are aged 30 y or more.	Australia
Lea 2018	Research: Review	Reuse of health data	Review of issues associated with the reuse of health data	Findings revealed four key challenges: (1) uncertain reliability of consent as a cornerstone of trust due to the limits to understanding and awareness of data sharing; (2) ethical challenges around equity and autonomy; (3) ambitious overly theoretical governance frameworks lacking practical validity; and (4) a clear desire for further public and individual engagement to achieve clearer and more nuanced knowledge dissemination around data sharing practice and governance frameworks.	UK
Lee 2014	Discussion/opinion	Using social media to detect people with mental health issues	Discusses the withdrawal of the Samaritan's Radar, a twitter‐based app to identify people at risk of suicide (no direct public engagement)	Argues that had the app been developed with the twitter community it would have been better received and more appropriate to user needs.	UK
Leorin 2019	Research: Co‐design	eHealth technologies for people with dementia	2 experts with dementia were invited to lead sessions for early career researchers at The Connected Health Summer School	Early‐stage researchers developed 6 app mock‐ups based on their discussions and co‐creation activities with the two experts with dementia. The reflections on this experience highlight positive learning experiences for researchers in eHealth and mHealth.	Italy
Liu 2016	Research: Algorithm development	Suicide prevention	3035 participants from US National Epidemiologic Survey on Alcohol and Related Conditions with suicidal ideation at their lowest mood at baseline were included to develop a risk algorithm.	The developed risk algorithm for predicting the recurrence of suicidal ideation has good discrimination and excellent calibration. Clinicians can use this algorithm to stratify the risk of recurrence in patients and thus improve personalized treatment approaches, make advice and further intensive monitoring.	USA
Lucas 2014	Research: quantitative research	Virtual humans in clinical interviews	239 participants interacted with a rapport‐building VH, some with a human interviewer, some with a computer.	Overall, this paper provides the first empirical evidence that VHs can increase willingness to disclose in a clinical interview context. Participants who believed they were interacting with a computer reported lower fear of self‐disclosure, lower impression management, displayed their sadness more intensely, and were rated by observers as more willing to disclose. These results suggest that automated VHs can help overcome a significant barrier to obtaining truthful patient information.	USA
Majeed‐Ariss 2019	Research: Systematic review	Apps for young people's mental health	Review of the literature on the effectiveness of mobile apps designed to support adolescents' management of their physical chronic or long‐term conditions. (no direct public engagement)	A key finding of the review is the paucity of evidence‐based apps (n = 4) that exist, in contrast to the thousands of apps available on the app market that are not evidence‐based or user or professional informed. Only 3 apps reported some form of public involvement.	UK
Martel 2019	Research: Design development	e‐Screening for mental health issues in young people	A bicultural mixed‐methods co‐design approach involving Māori youth. 3 phases over a 3‐y period will provide an iterative evaluation of the utility, feasibility, and acceptability of YouthCHAT, aiming to create a framework for wider‐scale roll‐out and implementation.	YouthCHAT has potential as a user‐friendly, time efficient, and culturally safe screening tool for early detection of mental health and risk behaviour issues in NZ youth. Involving input from community providers, users, and stakeholders will ensure that modifiable elements of YouthCHAT are tailored to meet the health needs specific to each context and will have a positive influence on future mental, physical, and social outcomes for NZ youth.	New Zealand
Martinez‐Martin 2018	Research: Review	Psychotherapy apps	Review of the ethical challenges presented by direct‐to‐consumer (DTC) digital psychotherapy services that do not involve oversight by a professional mental health provider (no direct public involvement)	In a DTC therapy app, there are no clear lines of accountability or associated ethical obligations to protect the user seeking mental health services. The types of DTC services that present ethical challenges include apps that use a digital platform to connect users to minimally trained non‐professional counsellors, as well as services that provide counselling steered by artificial intelligence and conversational agents. There is a need for adequate oversight of DTC non‐professional psychotherapy services and additional empirical research to inform policy that will provide protection to the consumer.	USA
Matthews 2016	Research: Co‐design and clinical testing	Serious mental illness	Co‐design of MoodRhythm, a smartphone and web app, with patients and therapists and a small clinical pilot with experienced IPSRT clinicians and patients with bipolar disorder	MoodRhythm uses the phone's onboard sensors to automatically track sleep and social activity patterns. Describes the role physiological computing could have not just in monitoring psychiatric illnesses according to existing broad categories of diagnosis but in helping radically tailor diagnoses to each individual patient and develop interventions that take advantage of idiosyncratic characteristics of each person's illness in order to increase patient engagement in and adherence to treatment.	USA
McCann 2008	Conference paper abstract	Active ageing	Design of smart textiles and wearable technologies for active ageing	Discusses the need for cross‐disciplinary collaborations and co‐design with older people needed for the design and development of smart textiles and wearable devices that promote optimal safe exercise to enhance the quality of life of the active ageing.	UK
McCauley 2019	Research: Evaluation of app	App for dementia and family carers	Evaluation of the usage of a reminiscence app by people living with dementia and their family carers.	The comparative analysis of the electronic event logs as ‘ground truth’ in combination with the qualitative lived experience can provide a deeper understanding on the usage of a reminiscence app for those living with dementia and their family carers. As such, this work also provides key insights into using mixed methods for evaluating human‐computer interaction technologies.	UK
McClelland 2018	Research: Participatory development project	Mental health app	Participatory design used four focus groups with mental health service users and clinicians to discuss utility, create a ‘mock up’ and test a prototype based on focus group feedback.	Key ideas emerging from the focus groups were adopted in the design of the app prototype: the use of colour to convey mood; simple mood tracking using familiar trigger icons; a calendar integrated with the service user's care plan; a help button linked to personal support; an avatar to personalize the app; and the inclusion of evidence‐based information.	UK
McCosker 2020	Research: Instagram hashtag analytics	Public engagement with mental health issues online	Analysis of public use and engagement with a hashtag (#depressed) on Instagram	Demonstrates that public use of hashtags on Instagram and other platforms can indicate how the public engage in diverse forms of engagement with mental health issues, events or collective experiences online.	UK
Mehmet 2020	Research: Stakeholder needs assessment and evaluation of intervention effectiveness	Equally Well—a programme to improve the physical health of people living with mental illness	Mental health consumers and carers engagement with a social marketing digital media strategy designed to support the implementation of a digital media, social marketing intervention to support those seeking to improve the physical health of people living with mental illness.	The strategy was developed using a co‐design methodology and provided links to self‐care resources, access to service providers, clinical tools for health professionals and links to existing successful rural programmes. Using a co‐design approach, the study demonstrated the potential of a social marketing digital media strategy as a health promotion methodology.	Australia
Merchant 2019	Research: Social media analysis	Detection of health conditions using social media	Analysis of social media content across approximately 20 million words written by 999 consenting patients.	This study, linking electronic medical record data with social media data from consenting patients, identified that patients’ Facebook status updates can predict many health conditions, suggesting opportunities to use social media data to determine disease onset or exacerbation and to conduct social media‐based health interventions.	USA
Meyer 2018	Research: User testing	Wearable mobile technologies	14 outpatients with a diagnosis of schizophrenia used a consumer wrist‐worn device and smartphone to continuously and remotely gather rest‐activity profiles over 2 mo	Extended use of wearable and mobile technologies is acceptable to people with schizophrenia living in a community setting. In the future, these technologies may allow predictive, objective markers of clinical status, including early markers of impending relapse.	UK
Miatello 2018	Research: Systems improvement	Data elicited through apps for health systems improvement	Experience‐based co‐design (EBCD) study involving youth with mental disorders (aged 16‐24), family members, and service providers to capture participant feedback on the myEXP apps.	Overall, the myEXP apps were more effective at eliciting experience data from youth compared with family members and service providers. Apps offer an appealing tool to elicit data from patients and family members who may feel stigma when receiving some services and a power imbalance when providing feedback to health‐care providers. Rich experience data were gathered from youth about treatment plans in real time through the apps. The apps also showed important promise as reflective tools for all participants. They may offer advantages in research that seeks to improve responsiveness in service delivery and build mutual understanding. The apps also offer choice in how data are elicited, encourage more candid feedback and help to overcome stigma, which are important considerations for some vulnerable populations. For service redesign research using approaches such as EBCD, apps offer real‐time data gathering that can complement and enhance traditional approaches such as retrospective interviews and observation.	Canada
Mikal 2016	Research: qualitative study	Ethical issues in using Twitter for population‐level depression monitoring	Investigates public attitudes (n = 26) towards utilizing public domain Twitter data for population‐level mental health monitoring using a qualitative methodology	Focus group data reveal a wide range of attitudes towards the use of public‐domain social media ‘big data’ in population health research, from enthusiasm, through acceptance, to opposition. Study results highlight new perspectives in the discussion of ethical use of public data, particularly with respect to consent, privacy, and oversight.	USA
Mizen 2019	Research: protocol for a longitudinal, population‐wide record‐linked natural experiment	Exposure to green blue spaces (GBS)for mental health	Tracking daily lived experience by linking GBS accessibility indices, residential GBS exposure and health data; to enable quantification of the impact of GBS on well‐being and common mental health disorders for a national population (Wales)	The study will use a Geographic Information System (GIS) to create quarterly household GBS accessibility indices and GBS exposure using digital map and satellite data for 1.4 million homes in Wales, UK (2008‐2018). It will link the GBS accessibility indices and GBS exposures to individual‐level mental health outcomes for 1.7 million people with general practitioner (GP) data and data from the National Survey for Wales (n=~12 000) on well‐being in the Secure Anonymised Information Linkage (SAIL) Databank.	UK
Murphy 2020	Research: Delphi exercise	digital mental health technologies	Service users with lived experience were involved as one stakeholder group in a Delphi exercise about digital mental health technologies	User involvement in the Delphi process allowed consensus to be achieved regarding the factors considered important for harnessing technology in primary and secondary mental health care. Knowledge of these factors can help users and providers of mental health services negotiate how best to move forward with digitally enabled systems of care.	UK
Ng 2018	Research: Interview study	Wearable technologies in post‐traumatic stress disorder	Veterans’ (n = 13) perspectives on Fitbit use in treatment for post‐traumatic stress disorder were captured in semi‐structured interviews after an intensive treatment programme for PTSD.	Veterans described three major motivations to use the Fitbit during their time in the programme: increase self‐awareness, support social interactions, and give back to other veterans. We also identified three major reasons certain features of the Fitbit were not used: lack of clarity around the purpose of the Fitbit, lack of meaning in the Fitbit data, and challenges in the veteran‐provider relationship.	USA
Nicholas 2015	Research: Systematic review	Mobile apps for bipolar disorder (BD)	Review of apps developed for people with BD	32 apps provided information and 50 were management tools including screening and assessment (n = 10), symptom monitoring (n = 35), community support (n = 4), and treatment (n = 1). Not even a quarter of apps (18/82, 22%) addressed privacy and security by providing a privacy policy. In general, the content of currently available apps for BD is not in line with practice guidelines or established self‐management principles. Apps also fail to provide important information to help users assess their quality, with most lacking source citation and a privacy policy.	Australia
Nielsen 2020	Protocol for a randomized controlled trial of a co‐produced, complex, health promotion intervention	Health promotion in women with prior gestational diabetes	Co‐produced multifaceted intervention for health improvement in 460 women at risk of type 2 diabetes and their families	Engagement of women, partners and families through participation in the research. Part of this complex intervention (Face‐it) is digital health coaching tailored to family needs. Outcome measures for the intervention include participant's quality of life and mental health status.	Denmark
Nurmi 2020	Research: App development and feasibility testing	Development of an app (Precious App)	The feasibility of the app was tested among 12 adults who were asked to interact with the prototype and think aloud. Semi‐structured interviews allowed participants to extend their statements. Participants’ interactions with the app were video recorded, transcribed, and analysed with deductive thematic analysis to identify the theoretical themes related to autonomy support and change talk.	Digitalized motivational interviewing using a smartphone app engages users in the behaviour change process. Engaging users in feasibility testing highlights how they perceive autonomy support in the app.	Finland
O’Connor 2020	Research: exploratory and descriptive account of a co‐design project	Co‐designing technology with people with dementia	Reflective evaluation drawing on participant perspectives (in‐depth interviews) with people with dementia, their carers, and others involved in co‐creating a mobile health application	The views of people with dementia, their carers, and project staff were similar regarding the complexity of the co‐design process, and the value the mobile app had for people with dementia and their families. Being involved in co‐production seemed to have numerous benefits for people with dementia and their carers as they gained new knowledge and skills, friendships, and a sense of achievement in creating a unique app that would benefit many people. The app also appeared useful in stimulating memory and cognitive function, aiding communication, and providing a sense of normalcy for people living with dementia and their carers.	UK
Ong 2020	Research: digital methods of patient feedback	Implementation of a particular digital feedback intervention that was co‐designed with health professionals and patients (the DEPEND study)	Qualitative data was collected through interviews and focus groups with professionals, patients and carers. In total 51 staff, 24 patients and 8 carers were included. Forty‐two observations of the use of the digital feedback system were carried out in the four settings. Data analysis was based on modified grounded theory and Normalization Process Theory (NPT) formed the conceptual framework.	Patients had a range of views depending on their familiarity with the digital world. Patients mentioned barriers such as kiosk not being visible, privacy, lack of digital know‐how, technical hitches with the touchscreen. Collective action in maintaining participation again differed between sites because of workload pressure, perceptions of roles and responsibilities; and in the mental health site major organizational change was taking place. For mental health service users, their relationship with staff and their own health status determined their digital use. The potential of digital feedback was recognized but implementation should take local contexts, different patient groups and organizational leadership into account. Patient involvement in change and adaptation of the intervention was important in enhancing the embedding of digital methods in routine feedback. NPT allowed for an in‐depth understanding of actions and interactions of both staff and patients.	UK
Ospina‐Pinillos 2019	Research: Participative qualitative study	Adapting a mental health eClinic	Participatory design methodologies with users (young people aged 16‐30 y, supportive others, and health professionals) to (1) conduct workshops with users to co‐design and culturally adapt the MHeC; (2) inform the development of the MHeC‐S alpha prototype; (3) test the usability of the MHeC‐S alpha prototype; (4) translate, culturally adapt, and face‐validate the MHeC‐S self‐report assessment; and (5) collect information to inform its beta prototype.	Through a research and development process, the researchers co‐designed and culturally adapted, developed and user tested, and evaluated the MHeC‐S. By translating and culturally adapting the MHeC to Spanish, they aimed to increase accessibility and availability of e‐mental health care in the developing world, and assist vulnerable populations that have migrated to English‐speaking countries.	Colombia
Ospina‐Pinillos 2020	Research: Community‐based participatory research	Health information technologies for mental health care	Using participatory design methodologies with Colombian end users (young people, their supportive others, and health professionals), this study aimed to conduct co‐design workshops to culturally adapt a Web‐based Mental Health eClinic (MHeC) for young people.	This community‐based participatory research involved the utilization of a research and development (R&D) cycle including 4 iterative phases: co‐design workshops; knowledge translation; tailoring to language, culture, and place (or context); and one‐on‐one user testing sessions. 2 co‐design workshops were held with 18 users‐young people (n = 7) and health professionals (n = 11). 10 users participated in one‐on‐one user testing sessions‐young people (n = 5), supportive others (n = 2), and health professionals (n = 3). Participants liked the idea of having an MHeC designed and adapted for Colombian young people, and its 5 key elements were acceptable in this context (home page and triage system, self‐report assessment, dashboard of results, booking and video‐visit system, and personalized well‐being plan). However, to be relevant in Colombia, participants stressed the need to develop additional functionality (eg, phone network backup; chat; geolocation; and integration with electronic medical records, apps, or electronic tools) as well as an adaptation of the self‐report assessment. Importantly, the latter not only included language but also culture and context.	Colombia
Paay 2018	Research: Cultural probe study	Interactive technologies for young adults with low self‐esteem	Cultural probe study with 11 young adults, including a focus group, to understand current practices in managing self‐esteem with everyday technologies. Co‐design of interactive digital artefacts for helping improve self‐esteem, to deploy as technology probes with 6 young adults for 4 wk.	Interactive technologies designed to help young people feel happier need to be flexible, adaptable, private, available, personalizable, and have an engaging form factor to inspire feelings of fondness towards having the device as part of their daily routines.	Australia
Padrez 2016	Research: Patient survey	Patient perspectives on sharing social media data	Adult Facebook/Twitter users who presented to an emergency department were queried about their willingness to share their social media data and EMR data with health researchers for the purpose of building a databank for research purposes.	Many patients are willing to share and link their social media data with EMR data. Sharing patients have several demographic and clinical differences compared with non‐sharers. A database that merges social media with EMR data has the potential to provide insights about individuals' health and health outcomes.	USA
Patel 2020	Review: Meta synthesis of qualitative literature on service user's views of DHIs	Digital health interventions for adults with depression	Systematic review of service users’ views and experiences regarding the acceptability and usability of DHIs for depression, anxiety, and somatoform disorders (no direct public engagement)	A total of 24 studies were included in the meta‐synthesis, and 3 key themes emerged with descriptive subthemes. The 3 key themes were initial motivations and approaches to DHIs, personalization of treatment, and the value of receiving personal support in DHIs. The meta‐synthesis suggests that participants’ initial beliefs about DHIs can have an important effect on their engagement with these types of interventions. Personal support was valued very highly as a major component of the success of DHIs. The main reason for this was the way it enabled individual personalization of care. The findings have implications for the design of future DHIs to improve uptake, retention, and outcomes in DHIs for depression, anxiety, and somatoform disorders.	UK
Peiris‐John 2020	Research: large‐scale secondary school health and well‐being survey	Adolescent mental health	Nine participatory, iterative co‐design sessions involving 29 adolescents, and two workshops with young people (n = 11), digital and health service providers (n = 11) and researchers (n = 9) to gain insights into end‐user perspectives on the concept and how best to integrate digital interventions in to the survey	Students perceived integrating access to digital health interventions into a large‐scale youth health survey as acceptable and highly beneficial. They did not want personalized/normative feedback, but thought that every student should be offered all the help options. Participants identified key principles: assurance of confidentiality, usability, participant choice and control, and language. They highlighted wording as important for ease and comfort, and emphasized the importance of user control. Participants expressed that it would be useful and acceptable for survey respondents to receive information about digital help options addressing a range of health and well‐being topics. The methodology of adolescent‐practitioner‐researcher collaboration and partnership was central to this research and provided useful insights for the development and delivery of adolescent health surveys integrated with digital help options. The results from the study provide useful data on the impact of digital health interventions integrated in large‐scale surveys, as a novel methodology.	New Zealand
Pennou 2019	Research: Review	Mobile interventions for people with dual diagnosis	Review of the literature on mobile interventions for mental health and addictions (no direct public engagement)	Mobile applications have the potential to assist people with dual disorders in using strategies between treatment sessions without having to rely on their memory.	Canada
Pera 2015	Research: Observational study of subjective well‐being (SWB)	Well‐being in co‐creation	Consumer‐grounded view (no direct public engagement)	The study measures how community affiliation, personal growth and utilitarian motives are predictors of subjective well‐being (SWB). The results illustrate that community affiliation and personal growth motives predict high scores of SWB, while utilitarian motives do not. In addition, empowered consumers who co‐create with others are happier than consumers who create alone. This indicates that direct interactions are not only a powerful platform for service co‐creation, but are also predictors of SWB. Traditional companies and decision makers can use benefits offered by digital fabrication services.	Italy
Pickersgill 2019	Research: Discursive	Digitization of psychiatry	Contextualizes and historicizes developments in digital technologies in psychiatry	Digital technologies have become part of normal practice and are seen by professionals as part of their professionalism and a way of producing knowledge about mental health conditions as one significant focus.	UK
Pickersgill 2019	Discussion paper	Access to psychological therapies	Discusses the English Improving Access to Psychological Therapies (IAPT) programme and the role of psychology in contemporary health care	Through IAPT, millions of citizens have encountered interventions such as cognitive behaviour therapy, largely for the treatment of depression and anxiety. This article interrogates how this national response to problems of mental ill health—and the problematization itself—was developed, accounted for, and sustained, by imbricating economic expertise with accounts of mental ill health and mechanisms of treatment.	UK
Pine 2020	Research: qualitative study	Computer video games (CVG) for young people's mental health	Student perspectives were captured using pen and paper feedback forms following a brief presentation to 13‐15‐y‐old adolescents in seven high schools (n = 207) followed by more detailed focus groups (n = 42) and workshops (n = 21) with interested students.	Participants reported playing CVGs several times a week or day to help relieve stress, feel more relaxed and relieve boredom. Most were also interested in the idea of a mental health CVG. Participants in focus groups and workshops confirmed that playing CVGs was common among themselves and their peers, and that the idea of a CVG with subtle and brief mental health content such as game‐linked 'micro messages' was appealing. Participants recommended that the game should have an engaging interface and subtle mental health skills and information.	New Zealand
Powell 2019	Research: Realist Evaluation (RE)	Young people with Attention deficit hyperactivity disorder (ADHD)	Interviews with key stakeholders (CAYP with ADHD, their parents and specialist clinicians)	Complex intervention development for complex populations such as children and young people with ADHD should adopt methods such as RE, to account for the context it is delivered in, and co‐design, which involves developing the intervention in partnership with key stakeholders to increase the likelihood that the intervention will succeed. The development of the guidelines outlined in this paper could be used for the future development of technologies that aim to facilitate self‐management in CAYP with ADHD.	UK
Pratt 2020	Discussion	Trust in mental health	Discussion of mental health movements and the role of health activists (no direct public engagement)	Draws together evidence and insights to explain how notions of truth have been contested by mental health activists to build trust in mental health care in the UK.	UK
Proudfoot 2010	Research: Consultation exercise	Acceptability of using mobile phones for mental health	Community consultations consisting of an online survey (n = 525), focus group discussions (n = 47), and interviews (n = 20)	Community attitudes towards the appropriation of mobile phones for the monitoring and self‐management of depression, anxiety, and stress appear to be positive as long as privacy and security provisions are assured, the programme is intuitive and easy to use, and the feedback is clear.	Australia
Realpe 2020	Research: Description of a co‐design project	Social cognition intervention for young people with psychosis	Co‐designed with service users, the researchers adapted existing manualized social cognition intervention for people with a first episode of psychosis to a virtual world environment. A group of young people who have used mental health services co‐designed a virtual environment to deliver an accessible social cognition intervention to a hard to engage service user group. An iterative process with young service users and the design team that included developing initial ideas, creating a prototype and testing the virtual world.	The co‐design process led to the development of a specific design, approach and protocol to be tested in a proof‐of‐concept trial. Young service users were integral to an agile and iterative design. The authors argue that technological innovations should be routinely co‐designed and co‐produced if they are to realize their potential to deliver acceptable and affordable mental health interventions.	UK
Robillard 2019	Research: Review of apps	Mental health apps	Review of mobile phone apps for mental health (no direct public engagement)	Results demonstrate that information collection is occurring with the majority of apps that allow users to track the status of their mental health. Most of the apps collected in the initial sample did not include a privacy statement. Findings raise concerns about consent, transparency, and data sharing associated with mental health apps and highlight the importance of improved regulation in the mobile app environment.	Canada
Satinsky 2018	Research: Qualitative research	Mental health service users’ perceptions of data sharing and data protection	Mental health service users’ perceptions regarding the current practice of administrative data‐driven research. A focus group using case study scenarios.	Participants were generally happy for data owners to share their health, social and economic data if the purpose was transparent and if the information would inform and improve health policy and practice. Participants were less keen on sharing data through digital applications.	UK
Sin 2019	Research: Conference abstract	eHealth for family carers of people with psychosis	Co‐producing and evaluating an innovative eHealth intervention for family carers of people with psychosis – the EFFIP Project. Participatory research activities involving key stakeholders and carers as end users to co‐design and co‐produce the eHealth intervention, using an agile build process. Further public involvement activities are integral to the on‐going project oversight and management of the evaluative study	Co‐production work helped optimize the intervention design. The researchers conducted a usability study on the prototype involving carers to test the delta‐build. These have led to the co‐production of an eHealth intervention (COPe‐support) providing information and psychosocial support for carers through the internet, promoting flexible access and individualized choices. The authors suggest the co‐production work has optimized the intervention design and usability fitting the end users’ needs and usage pattern in the real world.	UK
Sin 2019	Research: Co‐produced design and build study	eHealth intervention for family carers for people affected by psychosis	Participatory research methodologies were used to integrate public, patients, and carers perspectives into the eHealth intervention design and build process to improve the product's usability and acceptability.	The participatory research work led to the co‐production of an eHealth intervention (COPe‐support). The study methodology, results, and output have optimized the intervention design and usability, fitting the end users’ needs and usage pattern.	UK
Sin 2020	Study protocol for a randomized controlled trial (RCT)	Digital intervention for family carers for people affected by psychosis	Carers living in England are eligible participants if they provide at least weekly support to a family member or close friend affected by psychosis, and use internet communication (including emails) daily.	This RCT (COPe‐support) demonstrates how digital technologies are increasingly being used to support carer engagement in an RCT for a digital intervention. All trial procedures are run online, including collection of outcome measurements which participants will directly input into our secure platform.	UK
Strudwick 2020	Discussion: report from a multi‐stakeholder meeting	Advancing e‐mental health	The need for e‐mental health (electronic mental health) services was discussed at the 9th Annual Canadian E‐Mental Health Conference held in Toronto, Canada.	Given the large number of people requiring mental health care in Canada, this model of care delivery is not sufficient in its current form. E‐mental health technologies may offer an important solution to the problem. Themes that emerged from the discussions at the conference include (1) the importance of trust, transparency, human centredness, and compassion in the development and delivery of digital mental health technologies; (2) an emphasis on equity, diversity, inclusion, and access when implementing e‐mental health services; (3) the need to ensure that the mental health workforce is able to engage in a digital way of working; and (4) co‐production of e‐mental health services among a diverse stakeholder group becoming the standard way of working.	Canada
Sturt 2018	Research: Qualitative research	Digital consulting for young people living with long‐term conditions	Cognitive interviews with young people (n = 14) to assess the face and content validity of two patient‐reported outcome measures	Both young people and the clinicians found the research task complex. Young participants required considerably more researcher prompting to elicit examples related to digital consulting rather than their face‐to‐face care.	UK
Sylvia 2018	Research: web‐based participatory research	Patient centred research network	Through the PCORI Mood Network patients participate as collaborators in comparative effectiveness research and engage in all stages of research from setting priority questions, to governance and oversight of studies, to dissemination of results and feedback on services.	The ultimate goal of engaging patients in the Mood Network is to enhance their sense of empowerment and agency through collaboration with the research community. The Mood Network was constructed with experts by experience (patients), clinicians, researchers, and key advocacy partners. Participants can track their clinical progress, comment in blogs and forums, propose research questions and priorities, help evaluate treatments, and participate in future studies. Recruitment has been slower than anticipated, and participants are not as diverse as they could be. The issue of stigma around mood disorders needs to be placed centre‐stage.	
Tabba 2019	Conference paper	People with dementia (PWD) residing in long‐term care	Conference presentation discussing the use of virtual reality (VR) as a tool to provide 360°‐video based experiences for individuals with moderate to severe dementia residing in a locked psychiatric hospital.	The authors discuss at depth the appeal of using VR for PWD, and the observed impact of such interaction. They present the design opportunities, pitfalls, and recommendations for future deployment in health‐care services. This paper demonstrates the potential of VR as a virtual alternative to experiences that may be difficult to reach for PWD residing within locked setting.	UK
Tazawa 2020	Research: Test of wearable device	Wearable health devices	Forty‐five depressed patients and 41 healthy controls participated, creating a combined 5250 days' worth of data.	The results indicated that utilizing wearable devices and machine learning may be useful in identifying depression as well as assessing severity.	Japan
Terp 2016	Research: Participatory design project	Engaging young adults with schizophrenia in their care	A participatory design process, where young adults with schizophrenia (n = 4), health‐care providers (n = 7), software designers (n = 3), graphic designer (n = 1), graphic recorder (n = 1), and team leader (n = 1) co‐designed a smartphone application for use in early phase schizophrenia care.	Guided by Etienne Wenger's construct of Community of Practice, three major categories of characteristics and construction of a physical and relational environment supporting and inspiring participation and engagement were identified: (i) a pre‐narrative about a community of practice, (ii) the room for design is a community of practice and (iii) the community of practice as a practice of special qualities. It is concluded that participatory design can support and inspire participation and engagement in the development of mental health care with young adults with schizophrenia, given that the environment in which participatory design unfolds is transparent, flexible, secure and informal.	Denmark
Terp 2018	Research: Participatory qualitative research	Young adult's self‐management of schizophrenia	Participatory design thinking and methods to develop an app (MindFrame)	Young adults diagnosed with schizophrenia are amenable to use a smartphone app to monitor their health, manage their medication, and stay alert of the early signs of illness exacerbation.	Denmark
Thabrew 2018a	Research: Review	Principles of co‐design with children and young people	This review summarizes the applied core principles of co‐design and recommends techniques for undertaking co‐design with children and young people. (no direct public engagement)	Three examples of co‐design during the development of eHealth interventions ‐ Starship Rescue, a computer game for treating anxiety in children with long‐term physical conditions, a self‐monitoring app for use during treatment of depression in young people, and HABITS, the development of an emotional health and substance use app, and eHealth platform for young people, are provided to illustrate the value and challenges of this process.	New Zealand
Thabrew 2018b	Research: Protocol for a trial	eHealth intervention for young people with anxiety	Co‐design, development, and open trial of a prototype game‐based eHealth intervention to treat anxiety in young people aged 13‐18 with long‐term physical conditions (n = 48). A further 20 young people with long‐term physical conditions and anxiety will be recruited to participate in an open pilot trial to evaluate its acceptability, usability, and preliminary efficacy.	If acceptable and useful, this game‐based eHealth intervention may offer a cost‐effective and clinically useful intervention for addressing the psychological needs of over 16 000 young people with long‐term health conditions in New Zealand.	New Zealand
Thompson 2020	Research: Proof‐of‐concept trial	Early psychosis	Five groups of three to five individuals per group were recruited over 6 mo. Eight sessions of SCIT‐VR therapy were delivered through the virtual world platform.	The SCIT‐VR therapy delivered was feasible (36% consent rate and 73.3% intervention completion rate), acceptable (high overall post‐session satisfaction scores) and safe (no serious adverse events), and had high levels of participant satisfaction. Users found the environment immersive.	UK
Thorstad 2019	Research: Analysis of social media posts	Detection of mental illness	Language samples were collected from the social media website Reddit, drawing on posts to discussion groups focusing on different kinds of mental illness (clinical subreddits), as well as on posts to discussion groups focusing on non‐mental health topics (non‐clinical subreddits).	Words drawn from the clinical subreddits could be used to distinguish several kinds of mental illness (ADHD, anxiety, bipolar disorder, and depression). Interestingly, words drawn from the non‐clinical subreddits (eg travel, cooking, cars) could also be used to distinguish different categories of mental illness, implying that the impact of mental illness spills over into topics unrelated to mental illness. Most importantly, words derived from the non‐clinical subreddits predicted future postings to clinical subreddits, implying that everyday language contains signal about the likelihood of future mental illness, possibly before people are aware of their mental health condition. Finally, whereas models trained on clinical subreddits learned to focus on words indicating disorder‐specific symptoms, models trained to predict future mental illness learned to focus on words indicating life stress, suggesting that kinds of features that are predictive of mental illness may change over time.	USA
Torenholt 2020	Research: Qualitative research	Patient data work	Ethnographic fieldwork carried out among cancer patients receiving PRO (patient‐reported outcome) based follow‐up care	PRO patient data work as conceptualized as two simultaneous processes: the process of data filtering – patients filter information to fit the envisaged recipient and purpose; and the process of data sensing – patients evaluate their embodied experiences. Patients’ data work has implications beyond simply providing data that represent their experiences.	Denmark
Torous 2017	Opinion: Patient perspective	Patient‐driven innovation for mental health	The case is co‐written with an individual with schizophrenia, who openly shares his name and personal experience with mental health technology in order to educate and inspire others.	The authors argue that there is a clear advantage for patient voice to be heard: so we can all learn from their experiences at the direct intersection of mental health and technology innovation. This paper is the first in JMIR Mental Health's patient perspective series from those with lived experience.	USA
Torous 2019	Research: Review	Digital care tools for psychosis	Explores key challenges drawing on research evidence (no direct public engagement)	Improving understanding of and outcomes for early‐course psychosis (ECP) is a recognized global mental health priority. The authors argue digital health technologies can advance care for ECP by better accounting for clinical heterogeneity, offering better predictive models, increasing access to early interventions and enhancing existing treatment options.	USA
Torous 2019	Research: Development of sector guidelines for apps	Mental health apps	Development of standards and principles for the evaluation of mental health apps.	At a minimum, standards should include consideration of: a) data safety and privacy, b) effectiveness, c) user experience/adherence, d) data integration. Recommendations are made in these areas.	USA
Tucker 2019	Discussion and recommendations	Predictive models in suicide prevention	Discusses ethical considerations regarding the use of predictive models in suicide prevention clinical care.	Recommendations for navigating the ethical issues are provided as an initial framework for others who are considering the implementation of a predictive model to trigger suicide prevention initiatives.	USA
Turvey 2019	Research: Review	Electronic health records	Review of the evidence base on electronic health records (no direct public engagement)	Electronic health records combined with tethered patient portals now support a range of functions including electronic data capture of patient‐reported outcomes, trend reporting on clinical targets, secure messaging, and patient‐mediated health information exchange. The applications of these features require special consideration in psychiatric and behavioural health settings. Nonetheless, their potential to engage patients suffering from disorders in which passivity and withdrawal are endemic to their mental health condition, is great.	USA
Vacca 2019	Research: Co‐design	Emotional health communication support for parents and teens	Participatory design study with eight Latina teens, a tool and memes that challenge parental assumptions about Latina teen norms and behaviour were co‐designed.	Latina youth viewed technology as a tool for creative argumentation that enacted certain cultural practices. In turn, Latina parents reflected on the authoritative source of the memes reconciling the subjective argumentation of the teen voice with the perceived objectivity of open data incorporated into the memes. Drawing on theories of brokering and Latina emotional health, we present theoretical and design ideas for supporting Latinas to improve teen‐caregiver relationships through digital media creation platforms.	USA
van Os 2015	Research: Clinical study	Routine Outcome Monitoring (ROM)	34 patients with major depressive disorder, treated with antidepressants, the combined effect of treatment and natural course was examined over a period of 18 weeks with Ecological Momentary Assessment (EMA). EMA consisted of repeated, within‐subject, mini‐measurements of experience (eg positive affect, negative affect, medication side‐effects) and context (eg stressors, situations, activities) at 10 unselected semi‐random moments per day, for a period of 6 d, repeated three times over the 18‐week period (baseline, week 6 and week 18).	Novel mHealth technology makes it possible to collect real life, in‐the‐moment ambulatory data that allow for an ecologically valid assessment of personalized and contextualized emotional and behavioural adjustment in the flow daily life (mROM). This study supports the use of mROM as a means to involve the patient in the process of needs assessment and treatment. EMA data are meaningful to the patient, as they reflect daily life circumstances. Assessment of treatment response with mROM data allows for an interpretation of the effect of treatment at the level of daily life emotional and social adjustment – as an index of health, obviating the need for an exclusive focus on traditional measures of sickness.	The Netherlands
Vereenooghe 2019	Research: Quantitative evaluation	Digital well‐being interventions for people with intellectual disabilities	Digital intervention, developed with and for people with intellectual disabilities, to improve their subjective well‐being. Using a single‐group pre‐post design, 12 participants with intellectual disabilities and their caregivers completed the 4‐week intervention.	Participant acceptability of the intervention was high, and feedback covered multiple aspects of the intervention, including (1) programme concept and design, (2) programme content, and (3) intervention usage. The study shows people with intellectual disabilities and their caregivers are receptive to using digital well‐being interventions and such interventions are feasible in routine practice.	Germany
Villani 2017	Research: Review	Internet use by people with schizophrenia	Review of the literature on people with schizophrenia's use of the internet for mental health information (no direct public engagement)	People experiencing schizophrenia spectrum disorders or other psychotic disorders wish to find on the Internet trustful, non‐stigmatizing information about their disease, flexibility, security standards, and positive peer‐to‐peer exchanges. E‐mental health also appears to be desired by a substantial proportion of them.	France
Vugts 2020	Study protocol for a controlled trial and process evaluation	Serious gaming during multidisciplinary rehabilitation for patients with complex chronic pain or fatigue complaints	This controlled trial demonstrates patient engagement through embedded qualitative methods include unobtrusive collection and analyses of stakeholder focus group interviews, participant feedback and semi‐structured patient interviews.	An experimental group is composed of patients who follow serious gaming during an outpatient multidisciplinary programme at two sites of a Dutch rehabilitation centre. Selected participants will contribute their views and experiences about gaming as part of their care.	The Netherlands
Wang 2018	Research: Model development	Big data analytics	Model developed by analysing big data implementation cases (no direct public engagement)	In addition to conceptually defining four big data analytics capabilities, the model offers a strategic view of big data analytics. Three significant path‐to‐value chains were identified for health‐care organizations by applying the model, which provides practical insights for managers.	UK
Wang 2019	Research: Theory building	Theory‐driven user‐centric explainable AI (XAI)	Review of empirical application‐specific investigations of XAI exploring theoretical underpinnings of human decision making, drawing from the fields of philosophy and psychology. (no direct public engagement)	Proposes a conceptual framework for building human‐centred, decision‐theory‐driven XAI. Drawing on this framework, the authors identify pathways along which human cognitive patterns drives needs for building XAI and how XAI can mitigate common cognitive biases. They put this framework into practice by designing and implementing an explainable clinical diagnostic tool for intensive care phenotyping and conducting a co‐design exercise with clinicians.	Singapore
Wang 2020	Research: Review	Mental health apps	Review of recent literature on the efficacy of MH apps. (no direct public engagement)	There are ethical and practical considerations involved in using MH apps as an adjunct to telepsychotherapy. The authors propose recommendations for app evaluation, and provide an evaluation of 28 popular English‐language MH apps.	USA
Wangmo 2019	Research: Qualitative research	Intelligent Assistive Technology (IAT)	Explores the ethical issues of IATs in elderly and dementia care from a professional perspective (no direct public engagement)	Professional stakeholders find issues of patient autonomy and informed consent, quality of data management, distributive justice and human contact as ethical priorities. Divergences emerged in relation to how these ethical issues are interpreted, how conflicts between different ethical principles are resolved and what solutions should be implemented to overcome current challenges.	Switzerland
Waycott 2018	Conference workshop	Virtual reality for therapy	Conference workshop with professional/public audience	Virtual reality (VR) is now being designed and deployed in diverse sensitive settings, especially for therapeutic purposes. For example, VR experiences are used for diversional therapy in aged care and as therapy for people living with conditions such as phobias and post‐traumatic stress. While these uses of VR offer great promise, they also present significant challenges. Given the novelty of VR, its immersive nature, and its impact on the user's sense of reality, it can be particularly challenging to engage participants in co‐design and predict what might go wrong when implementing these technologies in sensitive settings.	Australia
Wentink 2019	Research: qualitative	eRehabilitation programmes in stroke care	End users were involved in six focus groups with patients/informal caregivers to identify user requirements	Requirements between stroke patients/informal caregivers and health professionals differed on several aspects. Therefore, involving the perspectives of all end users in the design process of stroke eRehabilitation programmes is needed to achieve a user‐centred design.	The Netherlands
Zhang 2019	Research: participatory research study	Development of an app for substance use disorders	A user‐oriented design approach, used three co‐design workshops 10 health care professionals and 10 patients to develop an app for substance use disorders	Patients critiqued the existing app, common issues identified were those of the design, visual probe task, and the included images. Outpatients were concerned with the safety of administration of the intervention. Inpatient participants recommended the addition of functionalities, such as information on the harms associated with the substance use, and for there to be enhancements in the design, images, and task. There were differences in opinion on the inclusion of gaming features, as only health‐care professionals endorsed their inclusion. The results from this research will guide the development of an app that meets the specific needs of patients and is still based on a pre‐existing validated task paradigm.	Singapore
Zhu 2020	Research: person‐centred design/model building	Wearable health monitoring products for elderly people	Older people were asked about their physical and psychological needs to inform design requirements and decision model for the design of wearable health monitoring products	Wearable health monitoring products should be based on a design model based on older people's perspectives about their physical and psychological needs	China

**Country:** We used the affiliation of the first author to classify the country of the article; however, several studies had international teams or were reviews of the global literature.

**Additional inclusions (title/abstract screening):** Trial protocols that demonstrate models of PPI in the concept, design, testing or implementation of AI technologies.

Reviews that synthesize findings relating to PPI but do not have any direct public engagement.

Discussion of theory/building or conceptual papers that seek to engage end users/patients but do not have direct public engagement.

**Additional exclusions (title/abstract screening):** Video or digital therapeutic or educational interventions or information giving that is not interactive.

Telemedicine (by telephone or video call).

Algorithms/development that are not computerized or digital data.

Online patient support groups that are used for online social support.
